# CD44 Plays a Critical Role in Regulating Diet-Induced Adipose Inflammation, Hepatic Steatosis, and Insulin Resistance

**DOI:** 10.1371/journal.pone.0058417

**Published:** 2013-03-07

**Authors:** Hong Soon Kang, Grace Liao, Laura M. DeGraff, Kevin Gerrish, Carl D. Bortner, Stavros Garantziotis, Anton M. Jetten

**Affiliations:** 1 Cell Biology Section, National Institutes of Health, Research Triangle Park, North Carolina, United States of America; 2 Microarray Group, National Institutes of Health, Research Triangle Park, North Carolina, United States of America; 3 Laboratory of Signal Transduction, Division of Intramural Research, National Institute of Environmental Health Sciences, National Institutes of Health, Research Triangle Park, North Carolina, United States of America; 4 Matrix Biology Section, LRB, National Institutes of Health, Research Triangle Park, North Carolina, United States of America; University College Dublin, Ireland

## Abstract

CD44 is a multifunctional membrane receptor implicated in the regulation of several biological processes, including inflammation. CD44 expression is elevated in liver and white adipose tissue (WAT) during obesity suggesting a possible regulatory role for CD44 in metabolic syndrome. To study this hypothesis, we examined the effect of the loss of CD44 expression on the development of various features of metabolic syndrome using CD44 null mice. Our study demonstrates that CD44-deficient mice (CD44KO) exhibit a significantly reduced susceptibility to the development of high fat-diet (HFD)-induced hepatic steatosis, WAT-associated inflammation, and insulin resistance. The decreased expression of genes involved in fatty acid synthesis and transport (Fasn and Cd36), *de novo* triglyceride synthesis (Mogat1), and triglyceride accumulation (Cidea, Cidec) appears in part responsible for the reduced hepatic lipid accumulation in CD44KO(HFD) mice. In addition, the expression of various inflammatory and cell matrix genes, including several chemokines and its receptors, osteopontin, and several matrix metalloproteinases and collagen genes was greatly diminished in CD44KO(HFD) liver consistent with reduced inflammation and fibrogenesis. In contrast, lipid accumulation was significantly increased in CD44KO(HFD) WAT, whereas inflammation as indicated by the reduced infiltration of macrophages and expression of macrophage marker genes, was significantly diminished in WAT of CD44KO(HFD) mice compared to WT(HFD) mice. CD44KO(HFD) mice remained considerably more insulin sensitive and glucose tolerant than WT(HFD) mice and exhibited lower blood insulin levels. Our study indicates that CD44 plays a critical role in regulating several aspects of metabolic syndrome and may provide a new therapeutic target in the management of insulin resistance.

## Introduction

Obesity is a major health-care concern in many Westernized countries. In the United States 30% of the population is considered obese, while more than 66% of adults and almost 17% of children and adolescents are overweight [1], [2]. Obesity highly increases the risk of several pathologies, including type 2 diabetes, cardiovascular disease, and nonalcoholic fatty liver disease (NAFLD). Underlying the etiology of these pathologies is a disruption of the integrated control of lipid and glucose homeostasis in several tissues, including, the liver, adipose tissue, muscle, and pancreas. Several mechanisms have been implicated in the development of insulin resistance. Abnormal accumulation of lipids in the liver has been reported to cause hepatic steatosis and accelerate the progression of insulin resistance [3], [4], [5]. This is supported by findings showing that deficiencies in a number lipid transport, lipogenic or lipolytic genes promote or inhibit the development of hepatic steatosis and insulin resistance. It is well recognized that obesity is associated with systemic low-grade inflammation and that this is an important contributory factor in the development of insulin resistance [4], [6], [7], [8]. Increased infiltration of proinflammatory M1 macrophages and other immune cells, including T lymphocytes, in adipose tissue and the subsequent release of proinflammatory cytokines, has been shown to play a critical role in this enhanced inflammatory state [9], [10], [11].

CD44 is a multifunctional cell membrane protein that can act as a receptor for hyaluronan and osteopontin (Opn; also named secreted phosphoprotein 1 or Spp1) [12], [13]. CD44 is expressed by most cells, including macrophages and hepatocytes, and has been implicated in many biological processes, including development, cancer metastasis, and cell adhesion [12], [14], [15]. CD44 has been reported to regulate a variety of inflammatory responses, including the induction of proinflammatory cytokines and the migration of macrophages and neutrophils [16], [17]. Binding of hyaluronan to CD44 can promote the interaction with a number of other cell surface proteins, for instance Tlr4 and EGFR, and influence the activity a variety of downstream protein kinase signaling pathways, including the MAP kinase and Akt pathways [13], [18]. A recent study reported an alternative mechanism of action that involved proteolytic cleavage of CD44, translocation of its intracytoplasmic domain to the nucleus, where it subsequently regulated the expression of the matrix metalloproteinase 9 gene (*MMP-9*) by interacting with DNA within its promoter region [19].

Recent studies showed that obesity-induced hepatic steatosis and WAT-associated inflammation are accompanied with increased CD44 expression and opened the possibility that CD44 might play a regulatory role in obesity and associated pathologies [20], [21]. To obtain further insights into the function of CD44 in metabolic syndrome, we compared the development of diet-induced hepatic steatosis, WAT-associated inflammation, and type 2 diabetes between wild type (WT) mice and mice deficient in CD44 expression (CD44KO). Our study demonstrates that CD44KO mice fed a high fat diet (HFD) are significantly less susceptible to developing hepatic steatosis, inflammation, and fibrogenesis. In addition, adipose tissue-associated accumulation of pro-inflammatory M1 macrophages was considerably less in CD44KO(HFD) compared to WT(HFD) mice and CD44KO(HFD) mice display greater insulin sensitivity and glucose tolerance. Taken together, our results suggest that CD44 may play a critical role in linking obesity to the development of insulin resistance by promoting hepatic steatosis and the infiltration of macrophages in adipose tissue. Because CD44 functions as a receptor, it might provide a convenient therapeutic target in the management of type 2 diabetes.

## Materials and Methods

### Experimental Animals

CD44-deficient mice were obtained from T. Mak [22] and backcrossed onto C57BL/6J for >10 generations. Male mice were supplied *ad libitum* with a normal chow NIH-A31 diet (ND). For diet induced-obesity (DIO) studies, 8–12 week-old male mice were fed a high fat diet (HFD: D12492, Research Diets Inc., New Brunswick, NJ) for 21 weeks, unless indicated otherwise. Adiposity was determined with a PIXImus densitometer (Lunar Corp., Madison, WI). All animal protocols followed the guidelines outlined by the NIH Guide for the Care and Use of Laboratory Animals and were approved by the Institutional Animal Care and Use Committee at the NIEHS.

### Histology and Immunohistochemistry

Liver, white and brown adipose tissue specimens (n = 4–6) were fixed in 10% neutralized buffered formalin (NBF), and embedded in paraffin. Five microns (5 µm) of tissue sections were stained with hematoxylin-eosin. In order to detect macrophages, sections of white adipose tissue (WAT) were stained with an F4/80 antibody (Santa Cruz, CA) and avidin-biotin-peroxidase detection system.

### Lipid and Glucose Analysis

Blood levels of glucose, cholesterol, triglycerides, and high-density lipoprotein (HDL) were analyzed as described previously [23] using the Cobas Mira Classic Chemistry System (Roche Diagnostics Systems Inc., Montclair, NJ). Serum insulin levels were analyzed with an insulin ELISA kit (Millipore, St. Charles, MO). To measure liver triglyceride and cholesterol contents, livers were homogenized and lipids extracted as previously reported [24]. Triglyceride and cholesterol levels were measured with Stanbio assay kits (Stanbio Laboratory, Boerne, TX).

### Microarray Analysis

Microarray analysis was performed using the Agilent 1-color protocol as described in previously [25] with RNA isolated from liver and WAT of WT(HFD) and CD44KO(HFD) mice (n = 5). The microarray data discussed in this report have been deposited in NCBIs Gene Expression Omnibus at http://www.ncbi.nlm.nih.gov/geo #GSE43104.

### RNA Isolation and Quantitative Real-time PCR (QRT-PCR)

Total RNA was isolated from liver and WAT with an RNeasy Mini kit from Qiagen (Valencia, CA). QRT-PCR analyses were performed as described previously [26]. QRT-PCR reactions were carried out in triplicate with StepOnePlus Real Time PCR system (Applied Biosystems). The sequences of primers and probes are listed in **Table S1**. All results were normalized relatively to the 18S or GAPDH transcripts. Student’s t-test was used to calculate the p-value.

### Western Blot Analysis

Protein lysates were prepared from WAT of mice fed a HFD using RIPA lysis buffer. Proteins were subsequently examined by Western blot analysis using antibodies against phosphorylated p38 MAP kinase (p-p38), phosphorylated JNK (p-JNK), and total p38 MAP kinase (p38) (Cell Signaling Technology).

### Isolation of the Stromal-vascular Fraction (SVF) and Flow Cytometry Analysis

The isolation of the SVF from epididymal WAT (eWAT) was carried out as described [23]. Macrophage and T lymphocyte populations were stained with fluorescent-conjugated monoclonal antibodies against F4/80, CD11b, CD11c, and CD206, or CD3, CD4, and CD8, respectively, and subsequently analyzed with a BD LSR II Flow cytometer (Becton Dickinson) using FACSDiVa software.

### Glucose Tolerance Test (GTT), Insulin Tolerance Test (ITT) and Adiponectin Elisa

After an overnight fast, WT and CD44 KO mice were injected intraperitoneally with glucose (2 g/kg) or insulin (0.75 U/kg) (Eli Lilly, Indianapolis, IN) and blood glucose levels determined to analyze GTT and ITT, respectively. A glucometer and glucose test strips (Nova Biomedical, Waltham, MA) were used to measure blood glucose. Circulating adiponectin levels were determined with an Elisa kit from R&D systems (Minneapolis, MN).

## Results

### Total Body Weight and Tissue Mass of CD44KO(HFD) Mice

**Figure 1:**
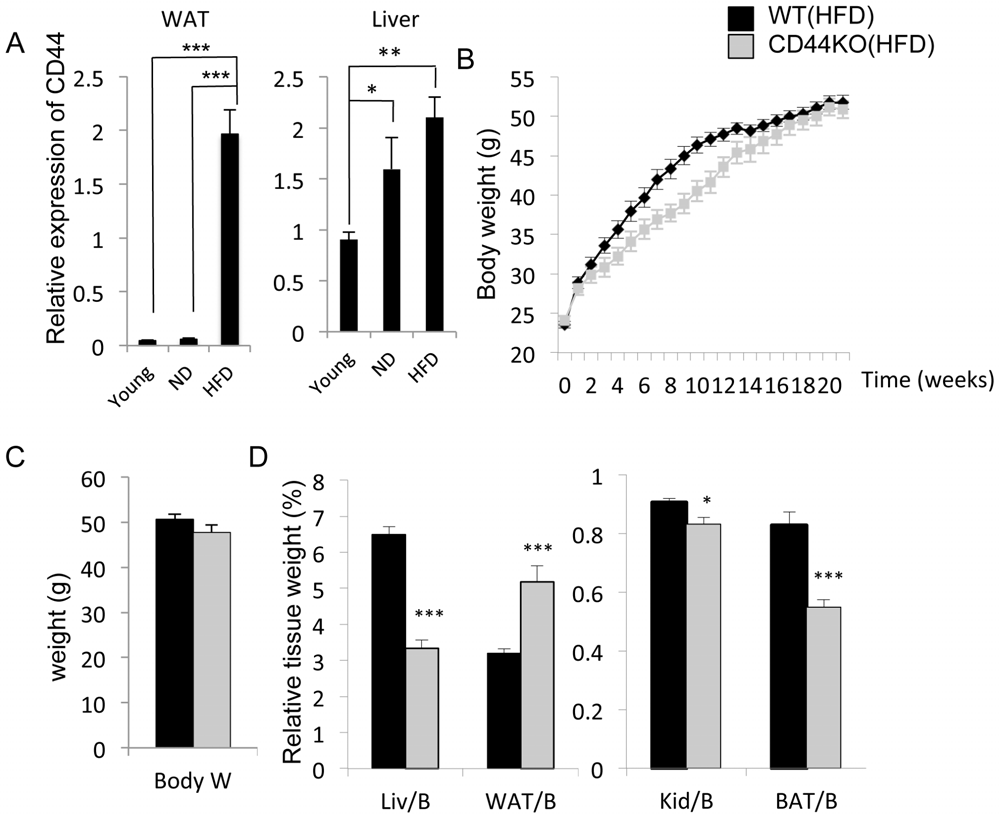
Comparison of total body mass and the relative tissue weight in CD44KO(HFD) and WT(HFD) mice. A) The expression of CD44 mRNA was analyzed in liver and WAT from 2 month-old WT (n = 6) or 7 month-old (n = 5) mice or mice fed a normal (ND) or HFD by QRT-PCR. B) Twelve week-old male mice (WT, n = 8; CD44KO, n = 8) were fed an HFD for 21 weeks and weight gain monitored. (C) Total body weight of mice fed an HFD for 21 weeks was plotted (WT, n = 8; CD44KO, n = 8). (D) Comparison of the relative weights of liver, WAT, kidneys, and BAT after 21 weeks on an HFD. Data present mean±SEM, *p<0.05, **p<0.01, ***p<0.001.

Recent studies showed a correlation between the level of CD44 expression and hepatic steatosis in obese patients [20]. Studies from our own laboratory revealed that CD44 was expressed at significantly higher levels in WAT of WT mice fed a HFD (**Fig. 1A**) and was also elevated in liver of 7 months old mice or mice fed a HFD compared to 2 months old mice. A similar correlation between CD44 expression and obesity was observed in WAT of WT mice and mice deficient in the nuclear receptor TAK1 (also called TR4) mice that are resistant to HFD-induced obesity (**Figure S1**) [23]. The association of obesity and increased expression of CD44 in liver and WAT suggested that CD44 might have a role in regulating certain aspects of metabolic syndrome.

To obtain further insights into the potential role of CD44 in metabolic syndrome, we monitored the development of diet-induced hepatic steatosis, adipose-associated inflammation, and type 2 diabetes in WT and CD44KO mice fed with a HFD ((WT(HFD) and CD44KO(HFD), respectively) for 21 weeks. CD44KO mice did not gain as much body weight as WT mice during the first 14 weeks on a HFD, while after 18 weeks the total body weights of WT and CD44KO mice were not significantly different (**Fig. 1B, C**). At 21 weeks, the relative weight of liver and brown adipose tissue (BAT) from CD44KO(HFD) mice was lower compared to WT(HFD) mice. In contrast, the relative weight of WAT was significantly higher (60%) compared to that of WT(HFD) mice, while the relative kidney weight was slightly decreased in CD44KO(HFD) mice (**Fig. 1D**). No significant difference in total body weight or the relative weight of WAT, kidney, and BAT was observed between 2 months old WT and CD44KO mice fed a normal diet, while the relative liver weight was slightly higher for CD44KO mice (**Figure S2**).

### CD44KO Mice are Protected against Diet-induced Hepatic Steatosis

**Figure 2:**
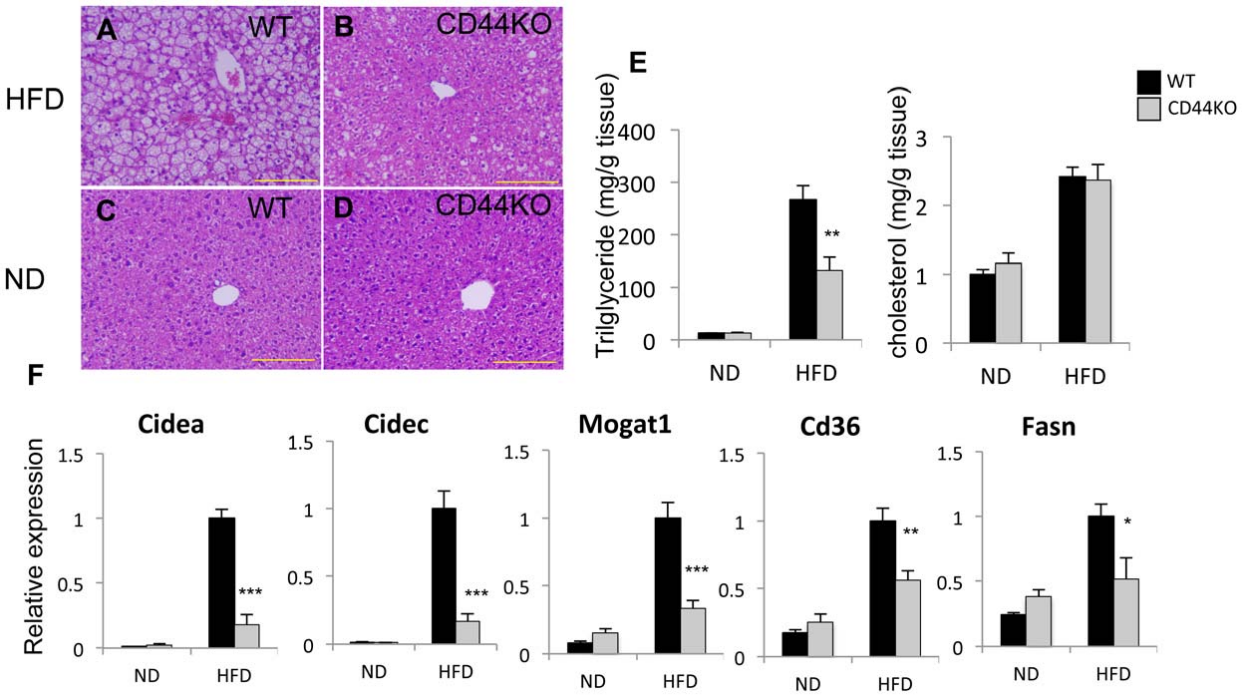
Hepatic steatosis was dramatically decreased in CD44KO(HFD). A–D) Representative H&E stained sections of liver were presented. Scale bar indicates 200 µm. E) Comparison of hepatic triglyceride and cholesterol levels. F) Comparison of gene expression in the livers of WT or CD44KO mice fed a normal diet (ND) or a high fat diet (HFD) (n = 5–6 mice per each group). Data present mean±SEM, *p<0.05, **p<0.01, ***p<0.001.

We next examined whether CD44-deficiency had any affect on the development of HFD-induced hepatic steatosis. Examination of histological sections of liver from WT(HFD) and CD44KO(HFD) showed a significant decrease in lipid accumulation in CD44KO(HFD) liver compared to WT(HFD) liver (**Fig. 2A, B**), while little difference was observed between livers of WT and CD44KO mice fed a normal diet (**Fig. 2C, D**). Lipid analysis indicated that hepatic cholesterol levels were increased to a similar degree in CD44KO(HFD) as in WT(HFD) mice (**Fig. 2E**). Although hepatic triglyceride levels were enhanced in both WT(HFD) and CD44KO(HFD) mice, this increase was about 50% less in CD44KO(HFD) consistent with the reduced fat accumulation observed in histological sections (**Fig. 2E**). The decrease in triglyceride accumulation in CD44KO(HFD) indicated that these mice were more resistant to the development of diet-induced hepatic steatosis.

The reduced accumulation of triglycerides is controlled at multiple levels, including fatty acid uptake, synthesis and storage of triglycerides, fatty acid oxidation, and lipolysis [4], [27]. To obtain greater insight into the mechanisms by which CD44KO mice are resistant to the development of hepatosteatosis, we compared the hepatic gene expression profiles between WT(HFD) and CD44KO(HFD) mice by microarray analysis (http://www.ncbi.nlm.nih.gov/geo; accession number GSE43104). Loss of CD44 function affected the expression of many genes that are implicated in lipid metabolism (**Table 1**). This analysis showed that several lipogenic genes, including Mogat1 and 2, Cd36, and Fasn, were expressed at significantly lower levels in CD44KO liver than in WT liver (**Table 1**). QRT-PCR analysis confirmed that the expression of Cd36, encoding a fatty acid transporter, and monoacylglycerol O-acyltransferase 1 (Mogat1), which is part of an alternative pathway of triglyceride synthesis, were expressed at 50–70% lower levels in CD44KO(HFD) liver than in WT(HFD) liver (**Fig. 2F**). The expression of Fasn, which is involved in *de novo* fatty acid synthesis, was also expressed at lower levels in CD44KO(HFD) liver. The expression of several other acyltransferases involved in triglyceride synthesis, including glycerol-3-phosphate acyltransferase (Gpat), acyl-glycerol-3-phosphate acyltransferases (Agpats), and diacylglycerol acyltransferase 1 and 2 (Dgat1 and Dgat2), was not significantly different between WT(HFD) and CD44KO(HFD) (**Figure S3A**). A dramatic decrease was observed in the induction of the cell death-inducing DFFA-like effectors a and c (*Cidea* and *Cidec*) in CD44KO(HFD) liver compared to WT(HFD) liver. Cidea and Cidec function as important regulators of energy homeostasis with roles in lipid storage and lipolysis [28]. Additional genes that are associated with triglyceride synthesis and were decreased in CD44KO(HFD) liver, included elongation of long chain fatty acids 5 and 7 (Elovl5 and -7), apolipoprotein a4 (Apoa4), perilipin 4 (Plin4), and lipin1 (Lpin1) (**Table 1**). No significant change was observed in the expression of several genes involved in lipid oxidation (Cpt-1α and Pparα), lipolysis (Hsl and Atgl), and gluconeogenesis (G6pase and Foxo1) (**Figure S3B**). These results indicated that the reduced hepatic steatosis in CD44KO mice is at least in part related to a diminished expression of a number of genes critically involved in the regulation of the uptake, synthesis, and accumulation of triglycerides rather than increased lipid oxidation, lipolysis, or increased gluconeogenesis.

**Table 1:** Partial list of genes reduced or increased in liver of CD44KO(HFD) mice compared to WT(HFD) mice.

Functional Category	GeneBankAccession #	Gene Symbol	Gene description		Fold Change (KO/WT)	
**Inflammation** **associated genes**						
*Chemokine*						
	NM_013654	Ccl7	chemokine (C-C motif) ligand 7		-5.23	
	NM_009987	Cx3cr1	chemokine (C-X3-C) receptor 1		−5.21	
	NM_013653	Ccl5	chemokine (C-C motif) ligand 5		−4.30	
	NM_009915	Ccr2	chemokine (C-C motif) receptor 2		−3.50	
	NM_013652	Ccl4	chemokine (C-C motif) ligand 4		−3.35	
	NM_008510	Xcl1	chemokine (C motif) ligand 1		−2.68	
	NM_009142	Cx3cl1	chemokine (C-X3-C motif) ligand 1		−2.42	
	NM_011888	Ccl19	chemokine (C-C motif) ligand 19		−2.32	
	NM_008599	Cxcl9	chemokine (C-X-C motif) ligand 9		−2.12	
	NM_007719	Ccr7	chemokine (C-C motif) receptor 7		−2.10	
	NM_009917	Ccr5	chemokine (C-C motif) receptor 5		−1.99	
	NM_007722	Cxcr7	chemokine (C-X-C motif) receptor 7		−1.98	
	NM_009141	Cxcl5	chemokine (C-X-C motif) ligand 5		−1.95	
	NM_001025192	Cxadr	coxsackie virus and adenovirus receptor		−1.68	
	NM_009910	Cxcr3	chemokine (C-X-C motif) receptor 3		−1.53	
	NM_018866	Cxcl13	chemokine (C-X-C motif) ligand 13		15.44	
*Immune response*						
	NM_009851	Cd44	CD44 antigen		−18.50	
	NM_010742	Ly6d	lymphocyte antigen 6 complex, locus D		−17.13	
	NM_001081110	Cd8a	CD8 antigen, alpha chain		−8.94	
	M17534	CD8b1	CD8 antigen, beta chan 1		−3.39	
	NM_001042580	Cd63	CD63 antigen		−3.19	
	NM_010555	Il1r2	interleukin receptor 1, type II		−1.71	
	NM_010741	Ly6c1	lymphocyte antigen 6 complex, locus C1		−1.65	
	NM_019408	Nfkb2	nuclear factor of κ light polypept. enhancer B cells		−1.55	
*Matrix*						
	NM_008607	Mmp13	matrix metallopeptidase 13		−11.90	
	NM_010809	Mmp3	matrix metallopeptidase 3		−4.54	
	NM_007742	Col1a1	collagen, type I, alpha 1		−4.37	
	NM_021334	Itgax	integrin alpha X		−4.03	
	NM_008605	Mmp12	matrix metallopeptidase 12		−3.62	
	NM_007737	Col5a2	collagen, type V, alpha 2		−3.06	
	NM_145467	Itgbl1	integrin, beta-like 1		−3.05	
	NM_009930	Col3a1	collagen, type III, alpha 1		−3.03	
	NM_020008	Clec7a	C-type lectin domain family 7, member a		−3.27	
	NM_011580	Thbs1	thrombospondin 1		−3.21	
	NM_007403	Adam8	a disintegrin and metallopeptidase domain 8		−3.19	
Functional Category	GeneBankAccession #	Gene Symbol	Gene description	Fold Change (KO/WT)		
	NM_001038604	Clec5a	C-type lectin domain family 5, member a	−3.08		
	NM_033612	Cela1	chymotrypsin-like elastase family, member 1	−2.68		
	NM_010705	Lgals3	lectin, galactose binding, soluble 3 (Mac-2)	−2.51		
	NM_008495	Lgals1	lectin, galactose binding, soluble 1	−2.35		
	NM_011693	Vcam1	vascular cell adhesion molecule 1	−2.24		
	NM_011581	Thbs2	thrombospondin 2	−2.12		
	AK037794	Itga4	integrin alpha 4	−2.05		
	NM_010810	Mmp7	matrix metallopeptidase 7	−1.70		
	NM_008611	Mmp8	matrix metallopeptidase 8	−1.62		
	AF026509	Itgb3	integrin beta 3	2.94		
	NM_010766	Marco	macrophage receptor with collagenous structure	7.73		
*Transcription*						
	NM_007498	Atf3	activating transcription factor 3	−2.80		
	NM_008091	Gata3	GATA binding protein 3	−1.69		
	NM_009283	Stat1	signal transducer and activator of transcription 1	−1.57		
	NM_011487	Stat4	signal transducer and activator of transcription 4	−1.57		
	NM_011756	Zfp36	zinc finger protein 36	1.89		
**Lipid accumulation associated genes**						
	NM_029001	Elovl7	elongation of long chain fatty acids, member 7	−9.83		
	NM_007468	Apoa4	apolipoprotein A-IV	−5.20		
	NM_007702	Cidea	cell death-inducing DFFA-like effector a	−4.42		
	NM_178373	Cidec	cell death-inducing DFFA-like effector c	−4.34		
	NM_027881	Osbpl3	oxysterol binding protein-like 3	−3.7		
	NM_020564	Sult5a1	sulfotransferase 5A1	−3.62		
	NM_177448	Mogat2	monoacylglycerol O-acyltransferase 2	−3.54		
	NM_020568	Plin4	perilipin 4	−2.67		
	NM_026713	Mogat1	monoacylglycerol O-acyltransferase 1	−2.39		
	NM_001033336	Abcc4	ATP-binding cassette, sub-family C, member 4	−2.00		
	NM_015763	Lpin1	lipin 1	−2.00		
	NM_007988	Fasn	fatty acid synthase	−1.82		
	NM_134255	Elovl5	elongation of long chain fatty acids, member 5	−1.70		
	NM_007643	Cd36	Cd36 antigen	−1.55		

**Figure 3:**
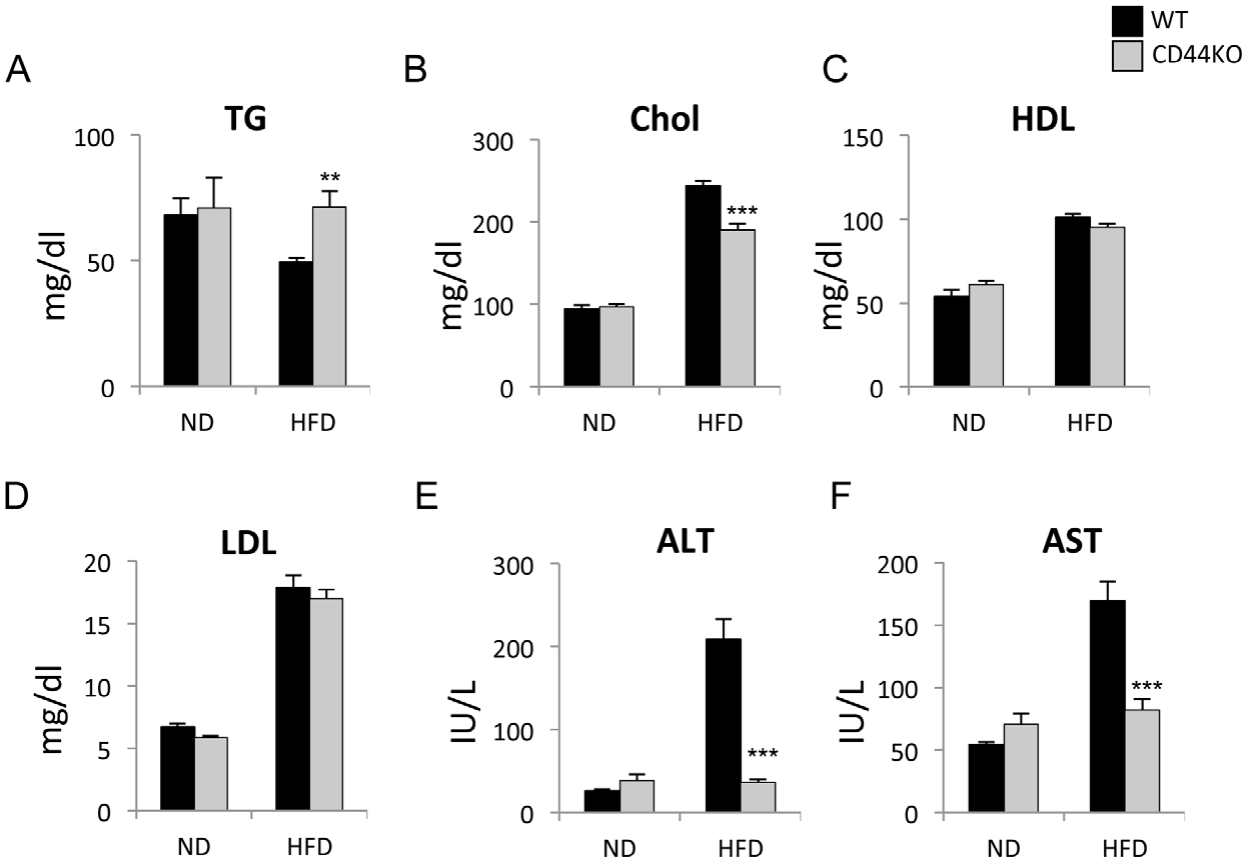
Activity of hepatic injury associated enzymes was decreased in CD44KO(HFD) compared to WT(HFD) mice. Levels of serum lipid (A–D), and alanine aminotransferase (ALT) and aspartate aminotransferase (AST) activity (E–F) were analyzed in WT and CD44KO mice fed a normal diet (ND) or a high fat diet (HFD) (n = 6–8 mice for each group). Data present mean±SEM, **p<0.01, ***p<0.001.

Biochemical analysis of sera from WT and CD44KO mice fed a ND or HFD showed that CD44KO(HFD) mice exhibited significantly lower serum cholesterol levels, while triglyceride levels were higher compared to WT(HFD) mice (**Fig. 3A, B**). The blood levels of high-density lipoprotein (HDL) and low-density lipoprotein (LDL) were increased under HFD conditions, but little difference was observed between the two genotypes (**Fig. 3C, D**).

### CD44KO Mice Display Reduced Hepatic Inflammation

Abnormal accumulation of lipids leads to NAFLD that encompasses various levels of severity of inflammation, hepatotoxicity, and fibrogenesis [29]. Analysis of blood levels of alanine aminotransferase (ALT) and aspartate aminotransferase (AST), biomarkers of hepatotoxicity, showed that their levels were dramatically increased in WT(HFD) mice compared to WT(ND), but were not significantly enhanced in CD44KO(HFD) mice (**Fig. 3E, F**). These data suggest that CD44KO(HFD) mice were considerably protected against the development of hepatotoxicity compared to WT(HFD) mice.

**Figure 4:**
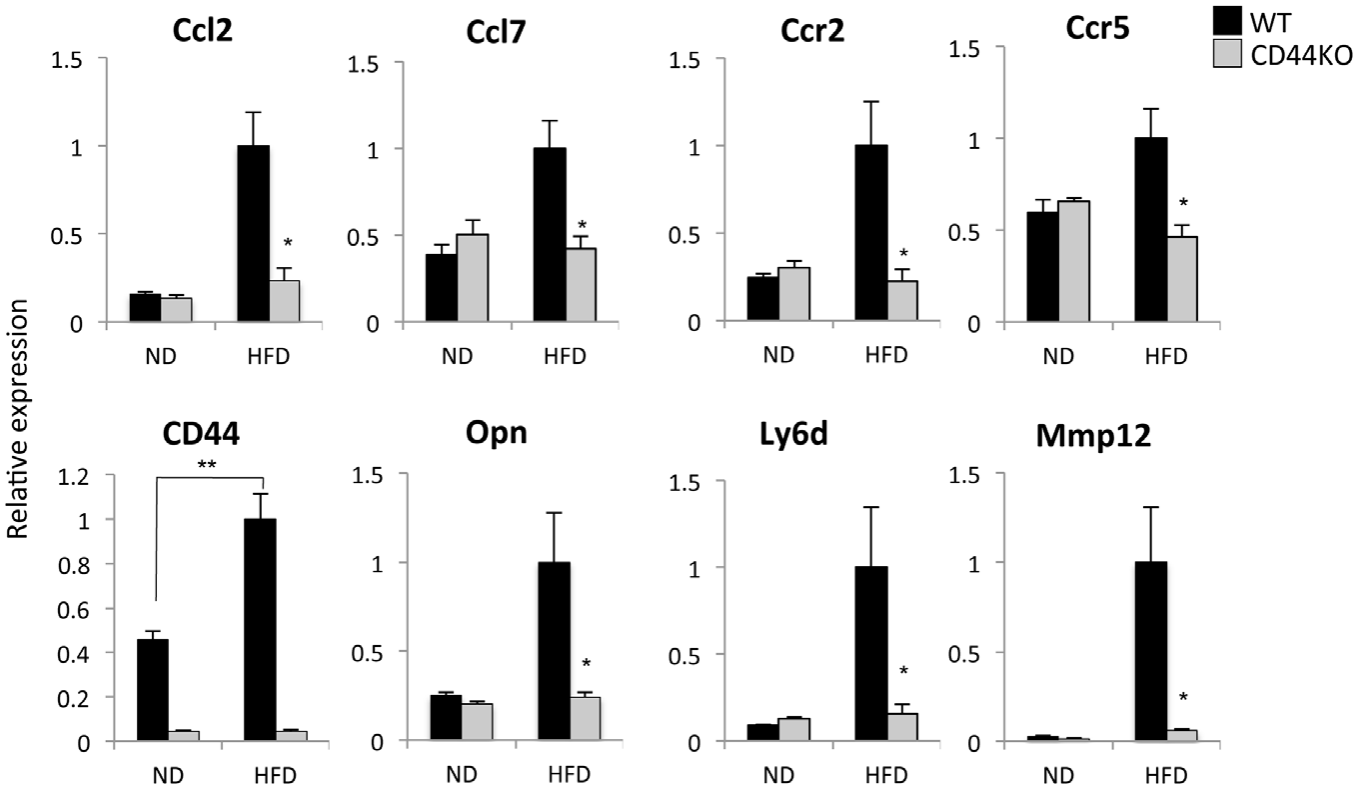
The induction of inflammatory genes was decreased in liver of CD44KO(HFD) compared to WT(HFD) mice. Comparison of gene expression in the liver of WT or CD44KO mice fed a normal diet (ND) or a high fat diet (HFD) (n = 5–6 mice per each group). Data present mean±SEM, *p<0.05, **p<0.01, ***p<0.001.

Our gene expression profiling further revealed that many genes encoding chemokines and their receptors were decreased in liver of CD44KO mice (**Table 1**). These included the chemokine (C-C motif) ligands Ccl4, 5, and 7, the chemokine (C-X3-C) receptor 1 (Cx3cr1), and the chemokine (C-C motif) receptors Ccr2 and Ccr7. The expression of several other genes associated with immune responses and inflammation, including the lymphocyte antigen 6 complex, locus D and C1 (Ly6d and Ly6c1), the interleukin receptor 1 type 2 (Il1r2), and the CD8 antigens α and β1 (Cd8a and Cd8b1), were decreased in CD44KO(HFD) liver (**Table 1**). QRT-PCR analysis confirmed that the expression of Ccl2, Ccl7, Ccr2, and Ccr5 were decreased in liver of CD44KO mice fed a HFD (**Fig. 4**). QRT-PCR analysis further confirmed the decreased expression of Ly6d in liver of CD44KO(HFD) mice compared to WT(HFD) mice consistent with a report showing that Ly6d expression positively correlates with hepatosteatosis (**Fig. 4**) [30]. The expression of the pro-inflammatory cytokine, osteopontin (Opn) and its receptor CD44, was also considerably induced in WT(HFD) liver in agreement with previous observations [20], [31], [32]. The induction of Opn was significantly diminished in CD44KO liver (**Fig. 4**). Together, these observations are consistent with the conclusion that CD44 deficiency in mice protects against hepatosteatosis-associated inflammation.

Inflammation and fibrogenesis are closely related during the progression of NAFLD [33]. Microarray analysis showed that a number of cellular matrix genes that are associated with increased fibrogenesis, including several collagen genes (Col1a1, 3a1, and 5a2) and matrix metalloproteinases (Mmp3, 12, and 13), were decreased in liver of CD44KO(HFD) mice (**Table 1**). These results suggest that CD44 deficiency in mice protects not only against inflammation, but also against the onset of fibrogenesis.

### CD44KO Mice Exhibited Increased Adiposity

**Figure 5:**
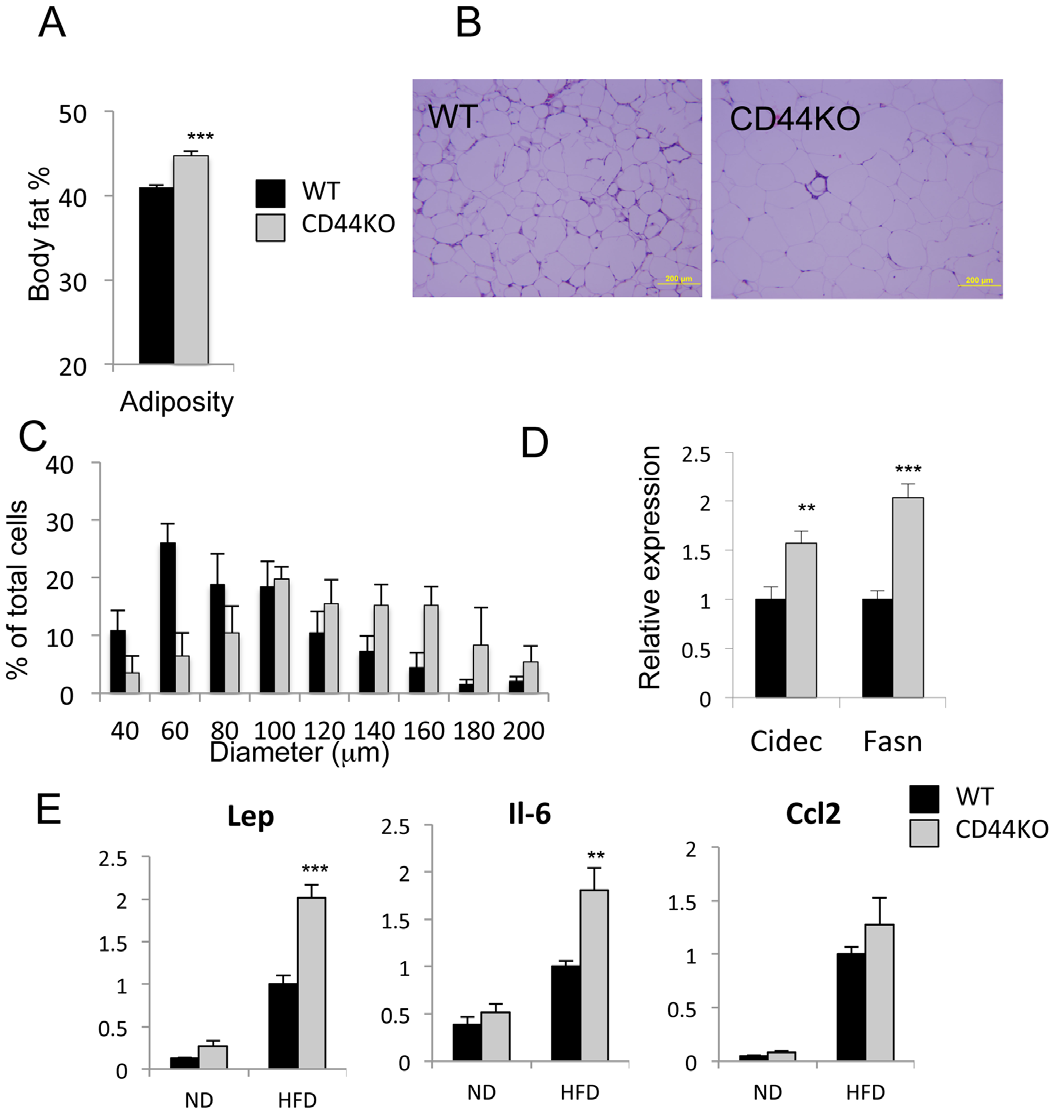
Total body fat was increased in CD44KO(HFD) compared to WT(HFD) mice. A) Total body fat mass from mice fed a HFD for 21 weeks was analyzed by Piximus densitometry. B) Representative H&E stained sections of WAT were presented. Scale bar indicates 200 µm. C) Comparison of the cell size of WAT adipocytes from WT(HFD) and CD44KO(HFD). Cell diameters in sections of WAT from 4 mice in each group were measured and the percentages of the different cell sizes calculated and plotted. D) Increased Cidec and Fasn expression in WAT of CD44KO(HFD) compared to WT(HFD) mice (n = 5–6 mice per each group). E) Comparison of gene expression in WAT of WT or CD44KO mice fed a normal diet (ND) or a high fat diet (HFD) (n = 5–6 mice per each group).

Examination of the total body fat percentage measured by PIXImus dual-energy X-ray absorptiometry, indicated that CD44KO(HFD) mice exhibited a significantly higher body fat index than WT(HFD) mice (**Fig. 5A**) consistent with the observed increase in the relative weight of epididymal WAT (**Fig. 1D**). In addition, the average size of CD44KO(HFD) adipocytes was larger than that of WT(HFD) adipocytes suggesting increased accumulation of lipids in CD44KO(HFD) adipocytes (**Fig. 5B, C**). The latter is supported by the increased expression of the lipogenic genes, Cidec and Fasn (**Fig. 5D**). Gene profiling analysis with WAT on WT(HFD) and CD44KO(HFD) mice identified a number of additional lipogenic genes, including Fabp1, Elovl6, and Mogat2, that were increased in CD44KO(HFD) WAT compared to WT(HFD) WAT (**Table** S**2**) consistent with the observed increased adiposity in CD44KO(HFD) mice. In contrast, no difference in the expression of the lipases, Hsl and Atgl, was observed. Previous studies reported increased expression of several adipokines, including leptin, Il-6, and Ccl2, in adipose tissues of obese mice [7], [34]. Similarly, the expression of leptin, Il-6, and Ccl2 was significantly increased in WAT of both WT(HFD) and CD44KO(HFD) mice; however, the increase in leptin and Il-6 expression was significantly greater in CD44KO(HFD) mice (**Fig. 5E**). No change in the level of expression of adiponectin, which plays a critical role in regulating inflammation and lipid metabolism and is down-regulated in obesity [35], was observed between WT(HFD) and CD44KO(HFD) nor in the circulating level of adiponectin (**Figure S4**).

### Adipose-associated Inflammation was Reduced in CD44KO Mice Fed with an HFD

**Figure 6:**
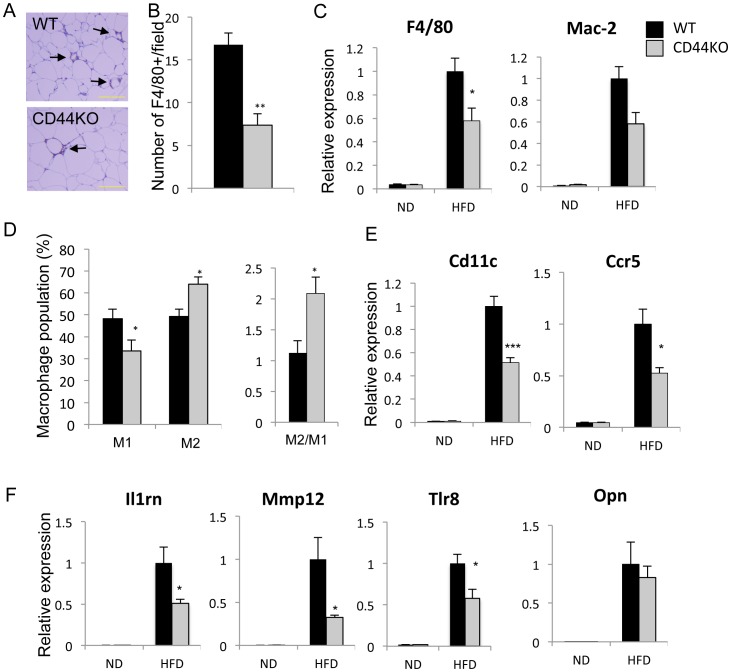
Adipose tissue inflammation was reduced in CD44KO(HFD) compared to WT(HFD) mice. A) Macrophages in crown-like structures (CLS) were identified by immunohistochemical staining with an F4/80 antibody as indicated by arrows. Scale bar indicates 250 µm. B) The number of CLS was decreased in CD44KO(HFD) compared to WT(HFD) mice. F4/80 positive cells in at least 4 randomly selected fields in sections from 4 different mice were counted. C) Comparison of the expression of macrophage markers, F4/80 and Mac-2, in WAT of WT or CD44KO mice fed a normal diet (ND) or a high fat diet (HFD) (n = 5–6 mice per each group). D) SVF cells from WAT of WT(HFD) mice (n = 6) and CD44KO(HFD) mice (n = 8) were examined by FACS analysis with F4/80, CD11b, CD11c, and CD206 antibodies. The percentage and ratio of pro-inflammatory macrophage (M1: F4/80^+^CD11C^+^CD206^−^) and anti-inflammatory macrophage (M2: F4/80^+^CD11C^−^CD206^+^) were determined. Expression of several macrophage markers (E) and inflammatory markers (F) was analyzed in WAT of WT or CD44KO mice fed a normal diet (ND) or a high fat diet (HFD) (n = 5–6 mice per each group).

Adipose tissue-associated inflammation, including increased infiltration of macrophages and T lymphocytes, has been reported to play a critical role in the development of obesity-linked pathologies, including insulin resistance [9], [10], [11]. Immunohistochemical analysis showed that compared to WT(HFD) mice, WAT of CD44KO(HFD) mice contained significant fewer crown-like structures (CLS), phagocytic macrophages surrounding dead adipocytes and adipocyte remnants (**Fig. 6A, B**). Moreover, QRT-PCR analysis demonstrated that the level of expression of the macrophage-specific markers, F4/80 (Emr1) and Mac-2, were significantly reduced in CD44KO(HFD) WAT (**Fig. 6C**). In addition, the ratio between alternatively activated (M2) macrophages (F4/80^+^CD11C^−^CD206^+^) and classical activated (M1) macrophages (F4/80^+^CD11C^+^CD206^−^) in WAT from CD44KO(HFD) mice was about 2∶1, compared to 1∶1 in WAT from WT(HFD) mice (**Fig. 6D**). The reduction in M1 macrophages was supported by the reduced expression of Cd11c in WAT of CD44KO(HFD) mice (**Fig. 6E**). WAT of both WT and CD44KO fed a ND consisted largely (90%) of M2 macrophages (**Figure S5**). To examine differences in immune responsiveness between WT and CD44KO macrophages, the response of peritoneal macrophages to LPS was compared; however, no significant differences were observed between the two genotypes in the induction of several inflammatory genes, including Ccl2 and Ccl5 (not shown).

Comparison of the gene expression profiles between WAT of WT(HFD) and CD44KO(HFD) showed that a number of inflammatory genes, including several cell surface antigens, tryptases and cyto/chemokines and their receptors, were down-regulated in CD44KO(HFD) WAT (**Table** S**2**). The observed decreased expression of several macrophage-associated genes, including interleukin 1 receptor antagonist (Il1rn), Mmp12, F4/80, Cd11c, Msr1, and Toll-like receptor 8 (Tlr8), supported the reduced macrophage accumulation in WAT of CD44KO(HFD) mice (**Table S2**; **Fig. 6F**). Although Opn expression was greatly induced in WAT of HFD mice, in contrast to liver no significant difference was observed between the two genotypes (**Fig. 6F**).

**Figure 7:**
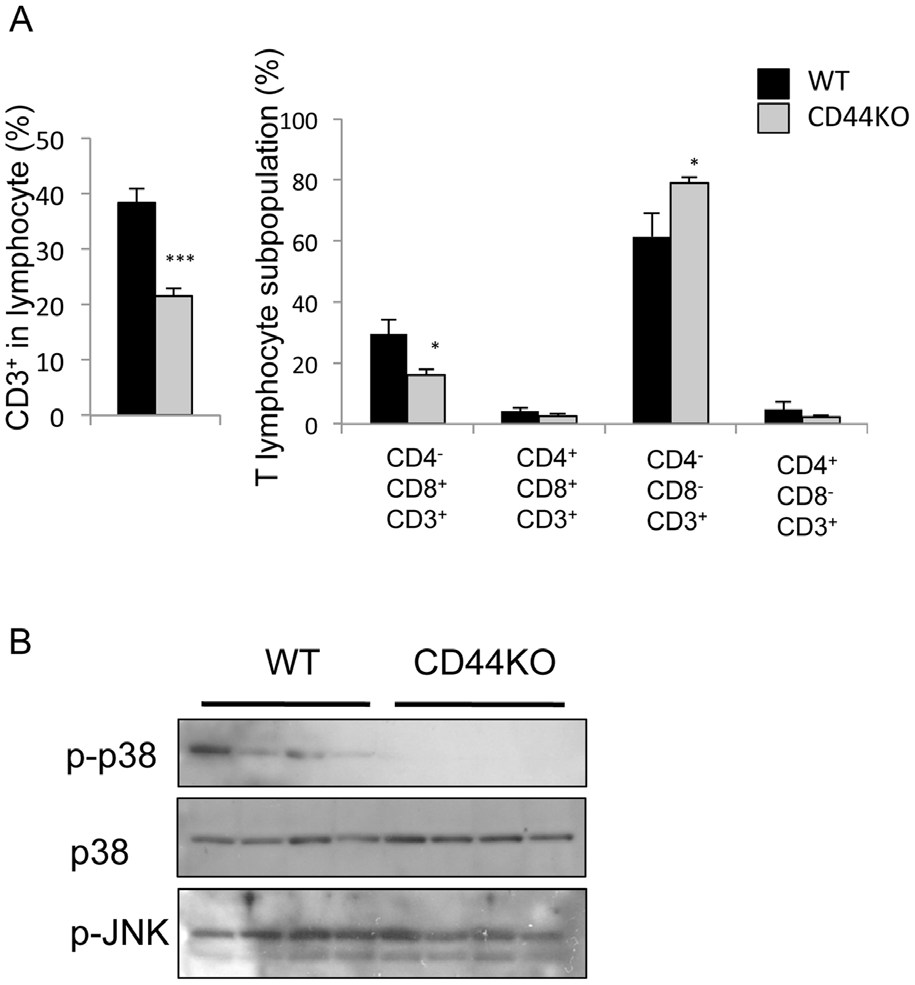
The CD3^+^ lymphocyte population and level of activated p38 MAPK in WAT of CD44KO(HFD) is reduced compared to WT (HFD) mice. The percentages of different CD4/CD8 cell populations were determined by FACS analysis. The percentage of CD8 single-positive T lymphocytes (CD4^−^CD8^+^) was decreased in WAT of CD44KO(HFD) compared to WT (HFD) mice. (n = 5–6 mice per each group). Data represent mean±SEM. *p<0.05, ***p<0.001. (B) Protein lysates were extracted from WAT of WT(HFD) and CD44KO(HFD) mice, and Western blot analysis was performed using phosphorylated or total p38 MAPK antibodies and phosphorylated JNK antibody.

Recent studies indicated that the accumulation of CD8^+^ T lymphocytes also play a critical role in WAT-associated inflammation [9]. **Fig. 7A** shows that the percent CD3^+^ T lymphocytes and CD8^+^ T lymphocytes was significantly decreased in WAT of CD44KO(HFD) compared to WT(HFD) mice consistent with reduced adipose-associated inflammation in CD44KO(HFD) mice. The decreased level of Cd3, Cd8a and Cd8b expression identified by microarray analysis (**Table** S**2**) is consistent with reduced infiltration of CD3^+^ and CD8^+^ cells. Together, these observations support the conclusion that adipose-associated inflammation is decreased in WAT of CD44KO(HFD) mice compared to WT(HFD) mice and is in part related to reduced infiltration and activation of M1 macrophages and CD8 lymphocytes.

Adiposity has been shown to be associated with activation of several kinases in WAT, including c-Jun amino-terminal kinases (JNKs) and p38 mitogen-activated protein kinase (p38 MAPK) [36], [37], [38]. Moreover, stimulation of CD44 signaling has been reported to activate p38 in different cell systems [39], [40]. We therefore examined the effect of loss of CD44 expression on the level of activation of these kinases in WAT by Western blot analysis. As shown in Fig. 7B, while phosphorylated JNK (pJNK) was unchanged, the level of phosphorylated p38 was dramatically decreased in WAT of CD44KO(HFD) mice compared to WT(HFD) mice. These results indicate that in WAT loss of CD44 results in a selective inhibition of the activation of p38 MAPK. Opn has been recently reported to activate p38 via CD44 [39], one might therefore speculate that activation of this pathway is impaired in CD44KO WAT.

### CD44 Mice are Protected against Diet-induced Insulin Resistance

**Figure 8:**
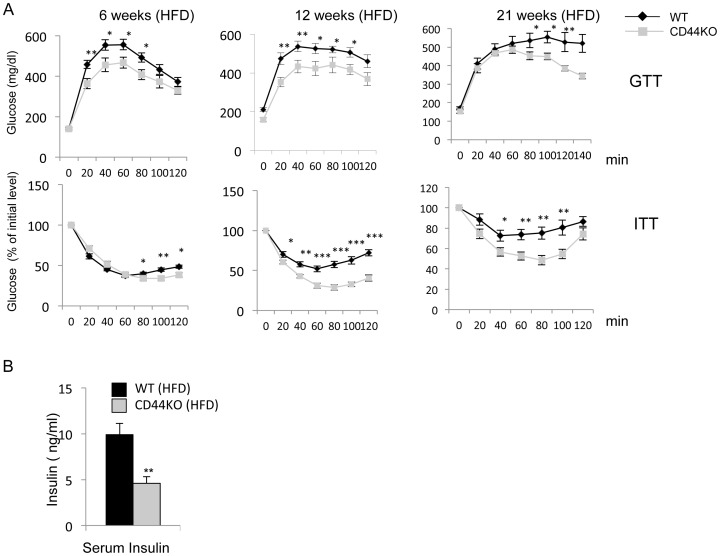
CD44KO mice are protected against HFD-induced insulin resistance and glucose intolerance. A) Glucose tolerance test (GTT) and insulin tolerance test (ITT) were analyzed in WT and CD44KO mice fed an HFD for 6 weeks, 12 weeks, or 21 weeks (n = 6–8 mice for each group). Blood glucose levels were analyzed every 20 min for up to 2 hrs after glucose or insulin injection, for GTT or ITT respectively. B) Blood insulin levels were analyzed in WT and CD44KO mice fed a HFD for 21 weeks (WT, n = 7; CD44KO, n = 8). Data represent mean±SEM. *p<0.05, **p<0.01, ***p<0.001.

Nonalcoholic fatty liver disease is almost invariably associated with insulin resistance [27]. To examine whether CD44KO(HFD) mice were less sensitive to the development of type 2 diabetes, glucose tolerance (GTT) and insulin tolerance tests (ITT) were performed at 6, 12, and 21 weeks of HFD feeding. As shown in **Fig. 8A**, at 6-weeks of HFD CD44KO mice were considerably more glucose tolerant than WT mice, while only a slight difference in insulin sensitivity was observed. At 12 weeks of HFD, CD44KO mice were significantly more glucose tolerant and insulin sensitive than WT mice. By 21 weeks of HFD, CD44KO mice remained considerably less glucose intolerance and insulin sensitive than WT mice (**Fig. 8A**). Insulin resistance often results in increased insulin production in an attempt to compensate for the decreased insulin sensitivity. As shown in **Fig. 8B**, serum insulin levels were significantly lower in CD44KO(HFD) mice than WT(HFD) mice. Together, these results indicate that CD44KO mice were less susceptible to the development of HFD-induced insulin resistance.

## Discussion

In this study, we examined the effect of CD44 deficiency on the development of diet-induced obesity and associated pathologies in mice. We demonstrated that CD44KO mice are protected against the development of diet-induced hepatic steatosis as indicated by the lower levels of hepatic triglycerides and reduced hepatotoxicity, inflammation, and fibrogenesis. In addition, our data showed that CD44KO mice are considerably less susceptible to the development of diet-induced adipose inflammation, insulin resistance, and glucose-intolerance.

**Figure 9:**
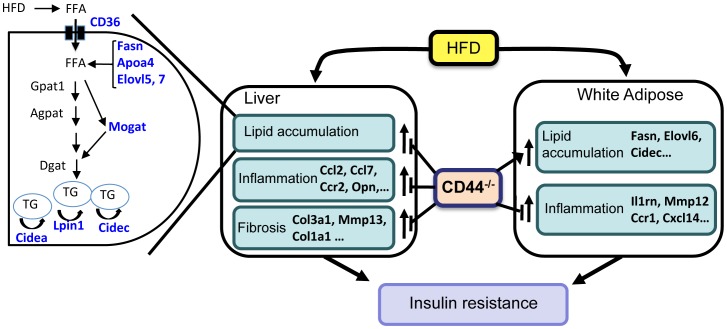
Schematic view of the links between CD44 deficiency and the development of diet-induced hepatic steatosis, inflammation, and fibrogenesis, adipose-associated inflammation, and insulin resistance. In liver, HFD induces the expression of many lipogenic genes, including Cd36, Fasn, Elovl, Apoa4, Mogat and Cidec and Cidea, which enhance the transport, synthesis, and accumulation of triglycerides. In addition, the expression of several inflammatory genes, such as Ccl2, Ccl7, Ccr2, and Opn, and fibrotic genes, including Col3a1, Mmp13, and Col1a1, are elevated and related to the observed increase in hepatic inflammation and the onset of hepatic fibrogenesis. In adipose tissue, HFD induces expression of several lipogenic genes and inflammatory genes (e.g., Il1rn, Mmp12, Ccr1, Cxcl14). The induction of these genes is in part responsible for the increased lipid accumulation and inflammation in WAT. Hepatosteatosis and WAT-associated inflammation subsequently promote the development of insulin resistance and glucose intolerance. The reduced expression of lipogenic, inflammatory, and fibrogenic genes in CD44-deficient mice is linked to their resistance to develop diet-induced hepatosteatosis. The inhibition of inflammatory genes and of the infiltration of macrophages and CD8^+^ lymphocytes in WAT of CD44KO mice results in reduced WAT-associated inflammation, while elevated expression of lipogenic genes is at least in part related to the observed increase in adiposity. The inhibition of hepatosteatosis and WAT-associated inflammation protects CD44-deficient mice against the development of insulin-resistance and glucose intolerance.

The accumulation of triglycerides during hepatic steatosis is mediated by several mechanisms, including changes in lipid transport, *de novo* synthesis, storage, fatty acid oxidation, and lipolysis. Our study demonstrated that the diet-induced increase in the expression of several genes implicated in the regulation of lipid metabolism was diminished considerably in the liver of CD44(HFD) mice. Of the genes analyzed, the expression of the cell death-inducing DFF45-like effector (CIDE) genes, Cidea and Cidec, was the most dramatically affected by CD44 deficiency (**Fig. 2**). Both Cidea and Cidec play a critical role in the regulation of lipid storage, lipid droplet formation, and lipolysis [24], [41], [42]. The expression of CD36, which among other things facilitates the transport of fatty acids into the liver, as well as the expression of several genes involved in fatty acid biosynthesis, including fatty acid synthase (Fasn), and Elovl5 and -7, were also significantly reduced in CD44(HFD) liver compared to WT(HFD) liver. Moreover, the expression of Mogat1, which is part of an alternative pathway of *de novo* triglyceride synthesis, was greatly diminished in CD44(HFD) liver, while the expression of Dgat1 and Dgat2, which are important in the classical *de novo* triglyceride synthesis pathway, was not different between the two genotypes. In addition to their control of lipid metabolism, several of these lipogenic genes, including Cidea, Cidec, and Cd36, play a critical role in regulating the development of hepatic steatosis and insulin sensitivity [24], [28], [43]. Mice deficient in Cidea or Cidec have been shown to exhibit reduced hepatic lipid accumulation and improved insulin sensitivity, while overexpression of Cidea in mouse liver promoted hepatic steatosis. Cd36 deficiency has been reported to protect mice against diet-induced hepatic steatosis and insulin resistance, while increased expression has the inverse effect [44], [45]. The reduced expression of Cidec, Cidea, Cd36, Mogat1, and Fasn, as well as Elovl5 and 7, may provide a mechanism for the diminished hepatic fatty acid uptake and triglyceride synthesis, and the decreased lipid accumulation and be at least in part responsible for the reduced hepatic steatosis and insulin resistance observed in CD44KO mice (Fig. 9).

In addition to triglyceride accumulation, inflammation, hepatotoxicity, and fibrogenesis are important features in the progression of NAFLD [33], [46], [47]. We provided evidence suggesting that hepatotoxicity was greatly reduced in CD44KO(HFD) mice compared to WT(HFD) mice as indicated by the significantly lower blood levels of the biomarkers, ALT and AST. We further observed that the liver of CD44KO(HFD) liver displayed less inflammation than WT(HFD) liver as indicated by gene expression profiling. The expression of a number of proinflammatory cytokines, including several chemokines (Ccl2, Ccl5, Ccl7) and their receptors (Ccr2, Ccr5), metalloproteinases, and Opn, was reduced in CD44KO liver. Several of these genes, including the Ccr2 and Ccr5 signaling pathways, have been implicated in the mobilization of monocytes [48]. These observations are consistent with previous reports showing a link between Opn expression and progression of hepatic injury and inflammation and an association between the level of Ccl2 expression and the severity of hepatosteatosis [20], [37], [49]. Ccl2 is produced by hepatocytes and hepatic macrophages and functions as a potent chemoattractive mediator for bone marrow-derived macrophages, while both Ccl2 and Opn can function as activators of M1 macrophages [20], [32], [50]. A strong link has been established between the regulation of inflammation and fibrogenesis [33]. The reduced hepatic expression of matrix metalloproteinases and several collagens in CD44KO(HFD) mice is consistent with the conclusion that these mice are protected against the onset of hepatosteatosis-associated fibrosis. The latter is consistent with recent findings on the role of CD44 in lung fibrosis [51]. Thus, the observed decreased expression of various chemokines and their receptors as well as several cellular matrix genes in CD44KO(HFD) mice might be at least in part responsible for the reduced hepatic inflammation injury, and fibrogenesis, and consequently for its protective effect against hepatosteatosis (Fig. 9).

In most mouse obesity models, the development of hepatic steatosis and increased adiposity are often associated [23]. Unexpectedly however, in contrast to the decrease in hepatic steatosis, lipid accumulation was enhanced in WAT CD44KO(HFD) mice, which appeared to be due to an increase in lipid storage. This may be in part related to the enhanced expression of Fasn, Elovl3 and -6, FABP1, and Cidec, a key factor regulating triglyceride storage and lipid droplet size in WAT [28], [41] rather than changes in lipolysis since the expression of the major lipases, Hasl and Atgl, was not significantly different between WAT of WT and CD44KO mice. As the expression of Pparγ was not different between genotype groups, increased lipid storage in CD44KO WAT may not be due to expansion or development of adipocytes (**Figure S4)**. Differences in adiposity might involve altered energy intake and expenditure; however, no significant differences in food intake, oxygen consumption, CO_2_ production, and heat generation were observed between WT and CD44KO mice (**Figure S6**). Thus, the effects on hepatic steatosis and adiposity appear to be related to an immune-mediated modulation of lipid metabolism, rather than changes in food intake or energy expenditure in CD44KO mice.

In contrast to adiposity, WAT-associated inflammation was significantly reduced in CD44(HFD) mice. This was indicated by gene expression profile analysis showing that a number of inflammatory genes, including Il17a, Il33, Il1rn, Ccrl1, and Ccl6, were significantly reduced in WAT of CD44KO(HFD) mice (**Table** S**2**). In addition, the number of macrophages associated with WAT of CD44KO(HFD) mice was significantly lower compared to WT(HFD) WAT as indicated by the presence of considerably fewer crown-like structures and significantly lower levels of expression of the macrophage markers, F4/80, Cd11c, and Mac-2. Furthermore, the expression of the cytokine receptor Ccr5, which plays a critical role in the recruitment and activation of WAT macrophages [52], was significantly lower in CD44KO(HFD) WAT than WT(HFD) WAT. Moreover, the ratio of anti-inflammatory M2 macrophages over pro-inflammatory M1 macrophages was significantly higher in CD44KO(HFD) WAT (**Fig. 6**). Together, these observations support the conclusion that chemotaxis as well as the activation of pro-inflammatory macrophages is significantly reduced in CD44KO(HFD) WAT. In addition to the reduction in macrophages, the number of cytotoxic CD8^+^ T lymphocytes, which play an essential role in the initiation and propagation of adipose inflammation [9], and the expression Cd3 and Cd8 antigens was significantly lower in WAT of CD44KO(HFD) mice suggesting a role for CD44 in regulating the migration and recruitment of these cells as well. It is well recognized that obesity is associated with low-grade systemic inflammation and that WAT-associated inflammation plays a key role in obesity-linked pathologies, including insulin resistance [53], [54]. Thus, the reduced inflammation observed in WAT of CD44KO(HFD) mice might be a part of the mechanism that protects these mice against insulin resistance and glucose-intolerance (Fig. 9).

Adipokines produced by adipocytes play a critical role in regulating inflammation, lipid metabolism as well as insulin resistance [35]. Adiponectin plays a protective role against inflammation by reducing expression of proinflammatory cytokines through inhibition of NF-κB signaling pathway [35], [55], [56] and regulate fatty acid oxidation in liver though activation of PPARα and adenosine monophosphate-activated protein kinase (AMPK). However, the expression of adiponectin in WAT as well as the circulating level of adiponectin were not changed between genotypes (**Figure** S**4**). This suggests that the protection against adipose-associated inflammation and hepatosteatosis in CD44KO(HFD) mice is not causally related to differences in adiponectin levels. Our observation that the level of expression of PPARα and genes involved in fatty acid oxidation was not significantly different between WT and in CD44KO liver is consistent with our data that adiponectin levels are unchanged between WT and CD44KO mice.

While this manuscript was in preparation, an expression-based genome-wide association (eGWAS) study linked *CD44* to type 2 diabetes in humans and reported that CD44-deficiency ameliorates insulin resistance in mice and humans [21]. Our study showing that CD44KO mice are protected against the development of diet-induced adipose inflammation and insulin resistance, is consistent with that report. However, our study provides additional insights into the crucial role of CD44 in diet-induced hepatic steatosis, inflammation, and fibrogenesis, and WAT- and liver-associated inflammation and suggests that migration and activation of inflammatory cells may be critical elements affected by the absence of CD44 (Fig. 9).

The precise mechanism by which this multi-functional protein regulates diet-induced inflammation and insulin resistance needs further study. CD44 might regulate the expression of certain lipogenic and inflammatory genes by a direct mechanism involving the interaction of its intracellular domain with respective promoter regions as recently reported for *MMP-9* [19]. It is intriguing that the pro-inflammatory cytokine Opn, which interacts with CD44, has also been reported to play a role in cell migration, macrophage activation, and inflammation in obesity [32], [57], [58]. Like CD44KO mice, Opn-deficient mice are protected against the development of HFD-induced hepatic steatosis, WAT-associated inflammation, and insulin resistance, while lipid storage in WAT is increased [31], [37]. Although Opn acts through several receptor mechanisms, one might hypothesize that the decreased susceptibility observed in both knockout mouse models might be due in part to disruption of the Opn-CD44 signaling pathway. This hypothesis is consistent with recent reports showing that Opn mediates obesity-induced migration and infiltration of macrophages in WAT [32], [59], [60] and this modulation appears to involve the CD44 pathway [61]. Therefore, one might hypothesize that the reduced hepatic steatosis and WAT-associated inflammation in CD44KO mice might be due in part to the inability of Opn to activate the CD44 pathway. It is interesting to note that obesity in humans and mice is associated with increased expression of Opn in both liver and WAT [20]. Our study shows that the expression of Opn was greatly repressed in liver of CD44KO(HFD) mice, but not in WAT. Thus, suppression of Opn expression in liver may provide an additional mechanism for the reduced susceptibility CD44KO(HFD) mice to hepatic steatosis and insulin resistance.

In summary, our study demonstrates that mice deficient in CD44 are considerably resistant to diet-induced hepatic steatosis, fibrogenesis, and inflammation, adipose-associated infiltration of M1 macrophages, glucose intolerance, and insulin resistance (Fig. 9). These observations suggest that CD44 provides a critical link between metabolic changes and the development of inflammation and insulin resistance. Because CD44 functions as a receptor, it may provide a convenient therapeutic target in the management of lipid dysregulation in diet-induced liver disease and type 2 diabetes.

## Supporting Information

Figure S1 **The expression of CD44 mRNA was analyzed in WAT from WT (n = 6) or orphan nuclear receptor TAK1 knockout (TAK1^−/−^) (n = 6) mice fed a HFD by QRT-PCR.**
(TIF)

Figure S2 **Comparison of the relative weights of liver, WAT, kidneys, and BAT of WT or CD44KO mice fed a normal diet (ND). (WT, n = 6; CD44KO, n = 6).** Data present mean±SEM, *p<0.05, **p<0.01, ***p<0.001.
(TIF)

Figure S3 **(A) The expression of genes involved in classical pathway of triglyceride synthesis Dgat1 and Dgat2 was not different in liver between WT(HFD) and CD44(HFD) mice.** (B) Comparison of gene expression in liver of WT or CD44KO mice fed a ND or an HFD. (n = 5–6 mice per each group). Data present mean±SEM, *p<0.05, **p<0.01, ***p<0.001.
(TIF)

Figure S4 **(A) Expression of adiponectin in WAT of WT or CD44KO mice fed a normal diet (ND) or a high fat diet (HFD) (n = 5–6 mice per each group) was analyzed by QRT-PCR.** (B) Circulating levels of adiponectin from sera were analyzed by an Elisa kit (R&D systems).
(TIF)

Figure S5 **Macrophage populations of SVF cells from WAT of WT(ND) and CD44KO(ND) mice (n = 6 mice per each group) were examined by FACS.** No difference was observed in the percentage of M1 and M2 macrophages between WT and CD44KO mice before HFD.
(TIF)

Figure S6 **Oxygen consumption, CO_2_ production, Heat generation, and respiratory exchange ratio (RER) were analyzed in WT(HFD) and CD44KO(HFD) mice (n = 6 mice per each group) with a LabMaster system (TSE systems INC, Chesterfield, MO).**
(TIF)

Table S1 **List of QRT-PCR primers.**
(DOCX)

Table S2 **Partial list of genes reduced or increased in WAT of CD44KO(HFD) mice compared to WT(HFD) mice.**
(DOCX)
