# Supplementary figures and images for: CD44 Plays a Critical Role in Regulating Diet-Induced Adipose Inflammation, Hepatic Steatosis, and Insulin Resistance
Source: PLoS One. 2013 Mar 7;8(3):e58417. doi: 10.1371/journal.pone.0058417 (PMC3591334; doi:10.1371/journal.pone.0058417)

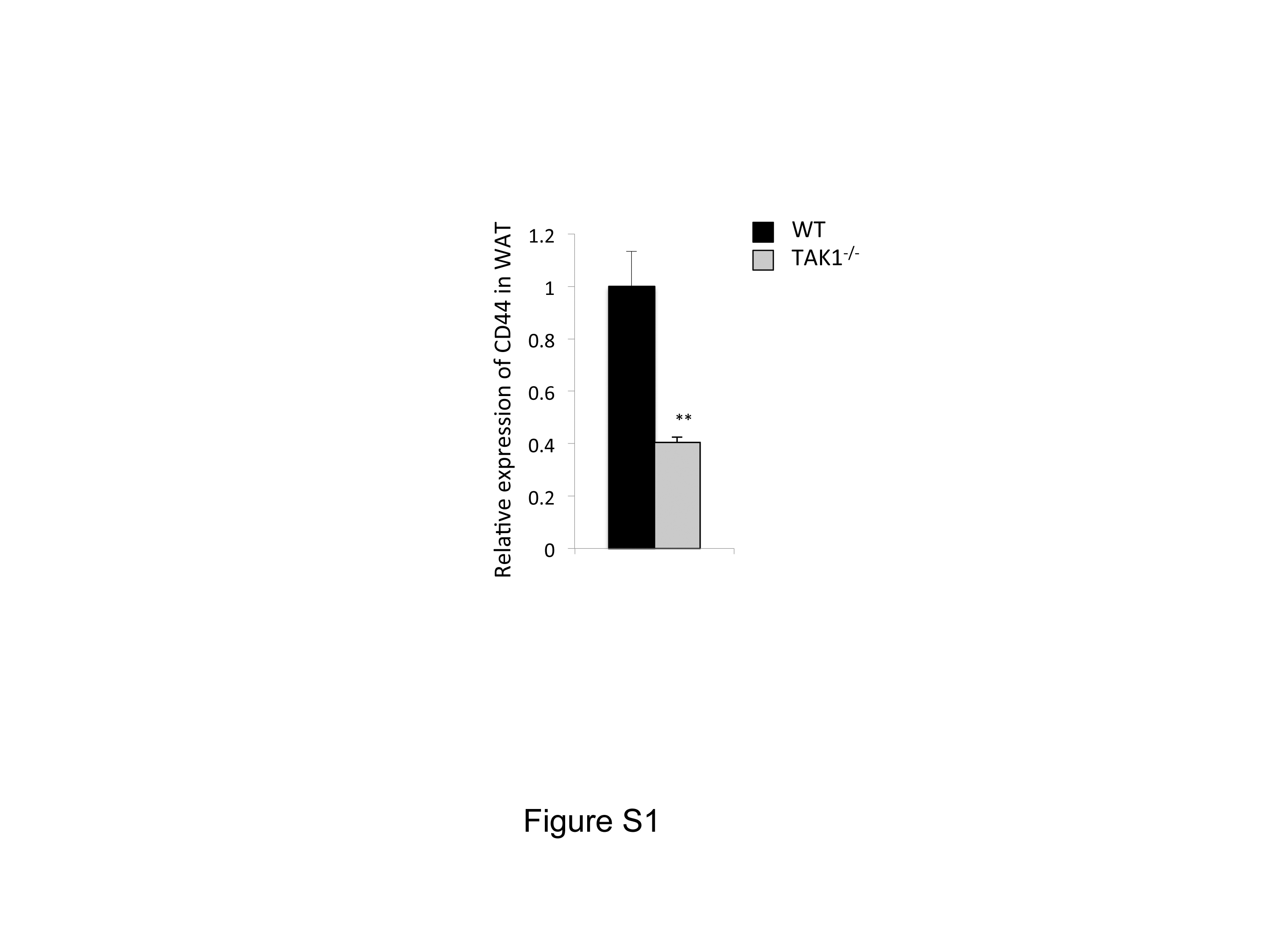

Supplement: Figure S1 — The expression of CD44 mRNA was analyzed in WAT from WT (n = 6) or orphan nuclear receptor TAK1 knockout (TAK1−/−) (n = 6) mice fed a HFD by QRT-PCR. (TIF) [file pone.0058417.s001.tif]

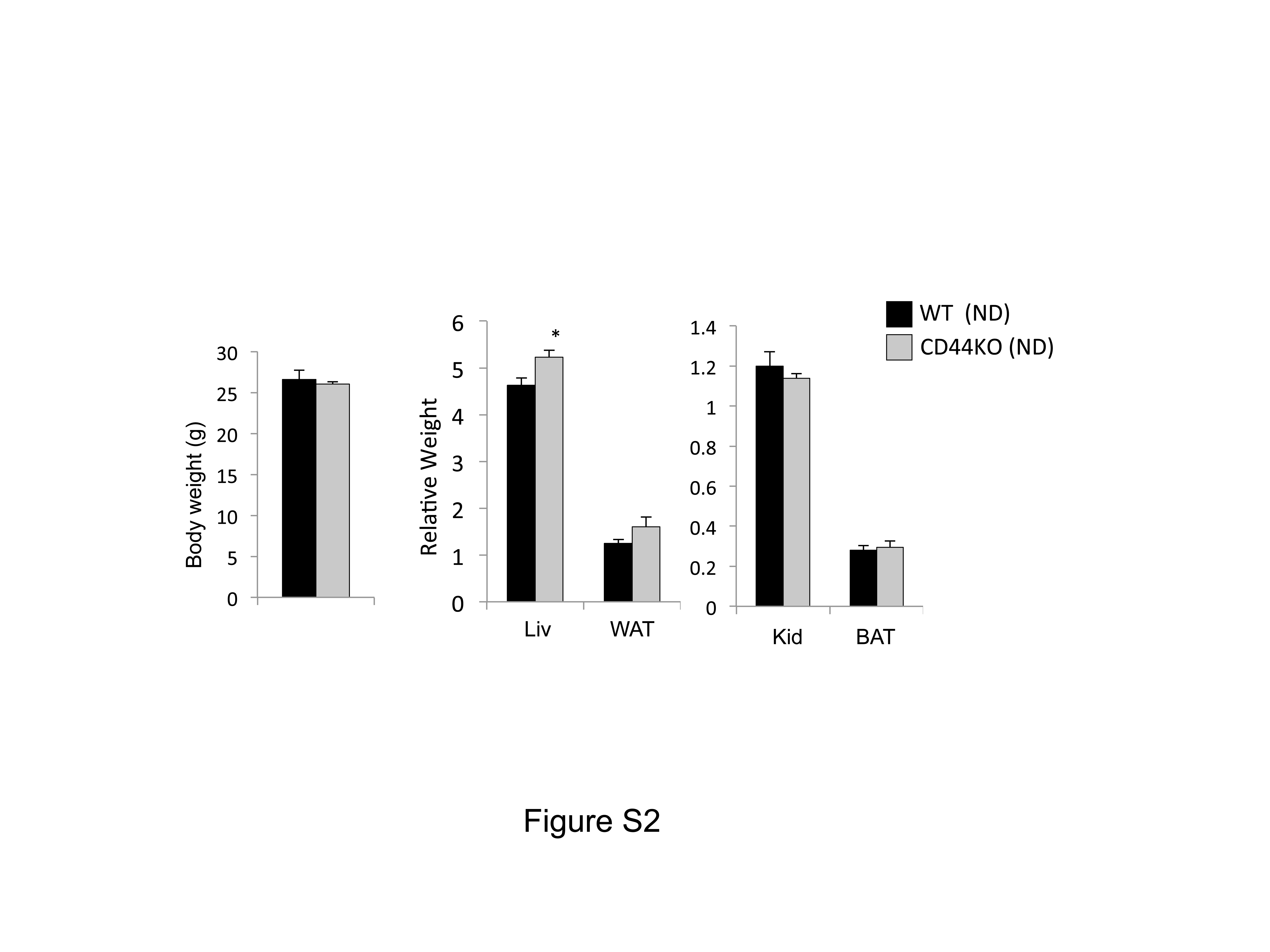

Supplement: Figure S2 — Comparison of the relative weights of liver, WAT, kidneys, and BAT of WT or CD44KO mice fed a normal diet (ND). (WT, n = 6; CD44KO, n = 6). Data present mean±SEM, *p<0.05, **p<0.01, ***p<0.001. (TIF) [file pone.0058417.s002.tif]

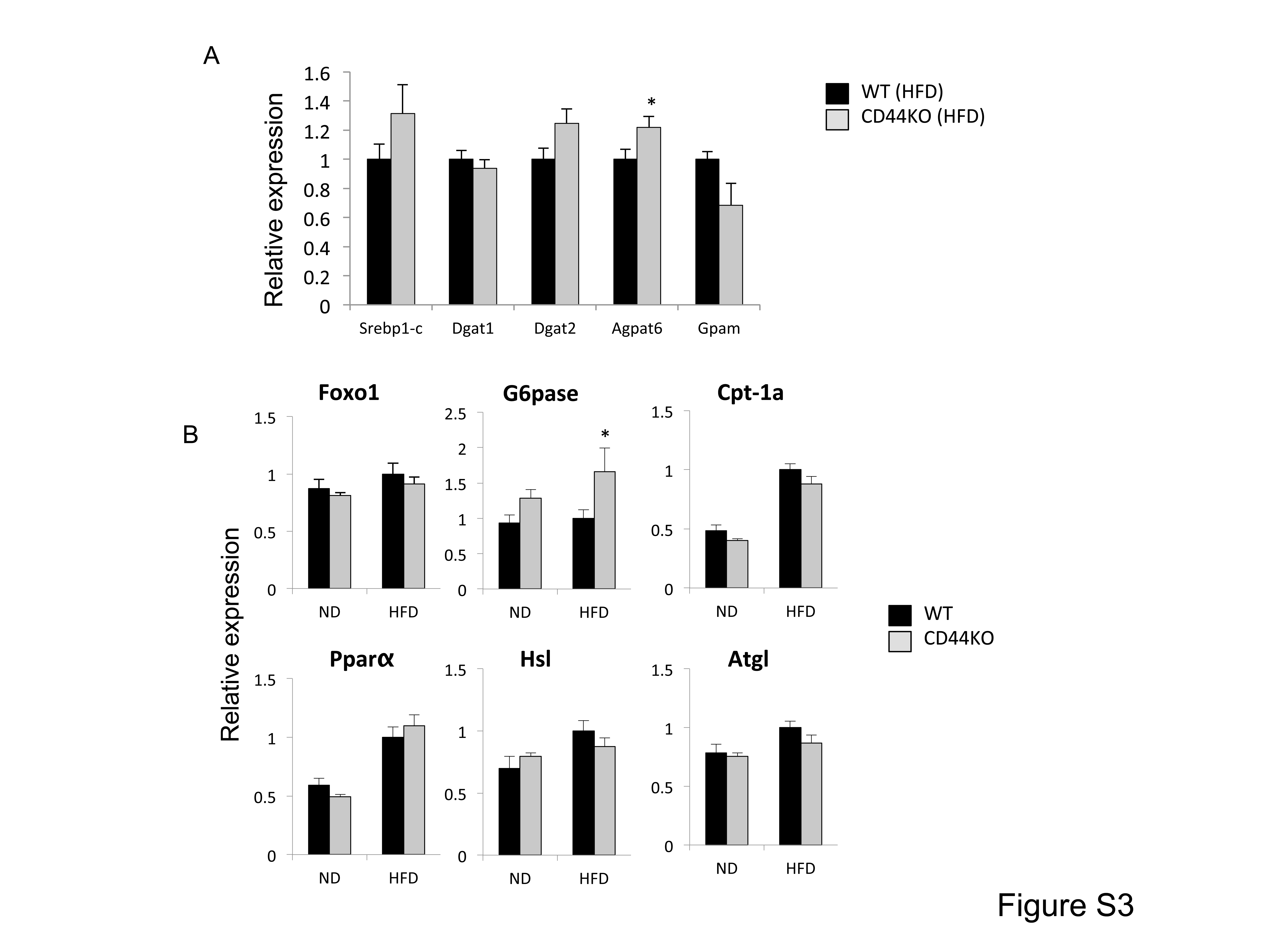

Supplement: Figure S3 — (A) The expression of genes involved in classical pathway of triglyceride synthesis Dgat1 and Dgat2 was not different in liver between WT(HFD) and CD44(HFD) mice. (B) Comparison of gene expression in liver of WT or CD44KO mice fed a ND or an HFD. (n = 5–6 mice per each group). Data present mean±SEM, *p<0.05, **p<0.01, ***p<0.001. (TIF) [file pone.0058417.s003.tif]

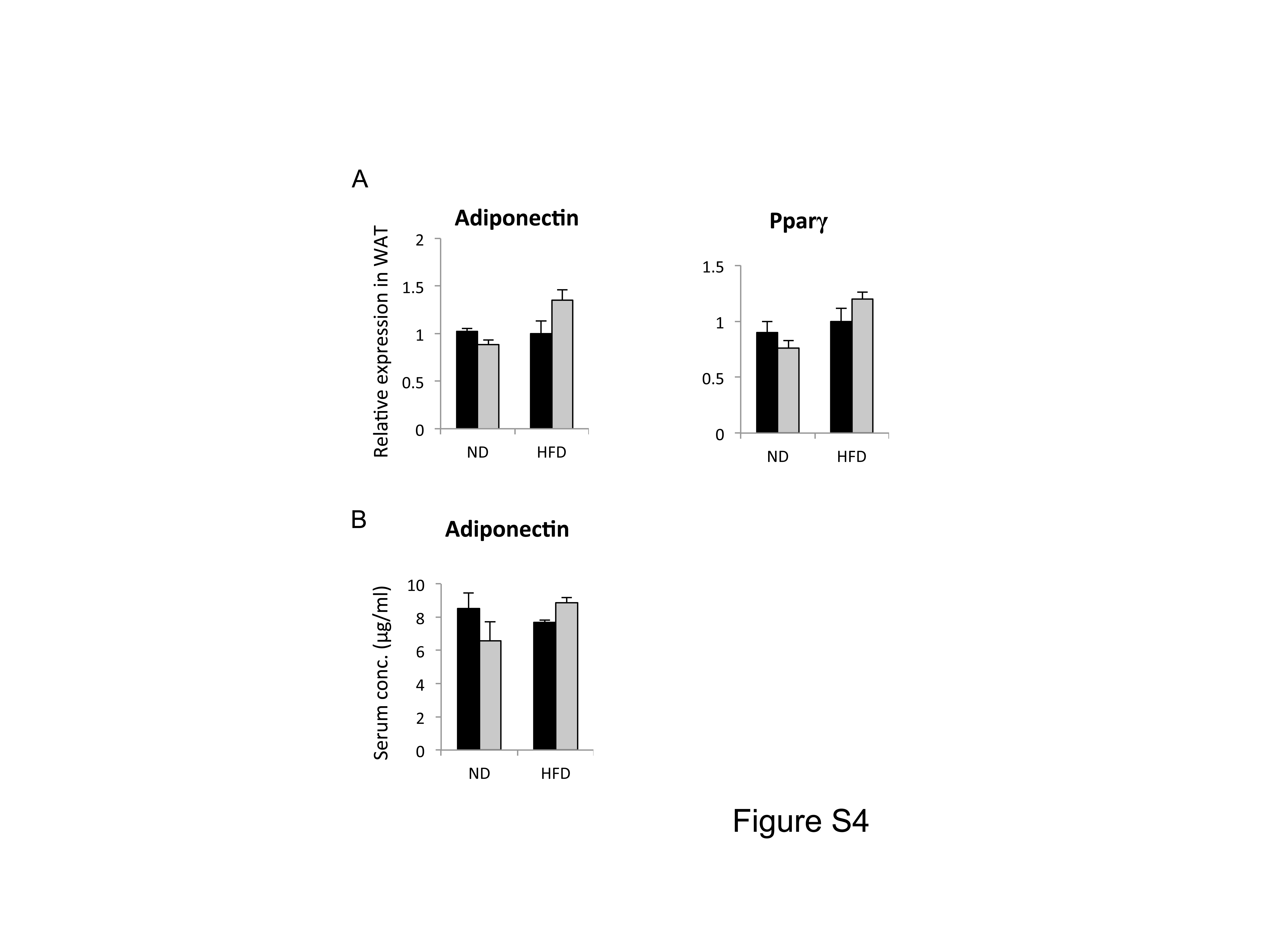

Supplement: Figure S4 — (A) Expression of adiponectin in WAT of WT or CD44KO mice fed a normal diet (ND) or a high fat diet (HFD) (n = 5–6 mice per each group) was analyzed by QRT-PCR. (B) Circulating levels of adiponectin from sera were analyzed by an Elisa kit (R&D systems). (TIF) [file pone.0058417.s004.tif]

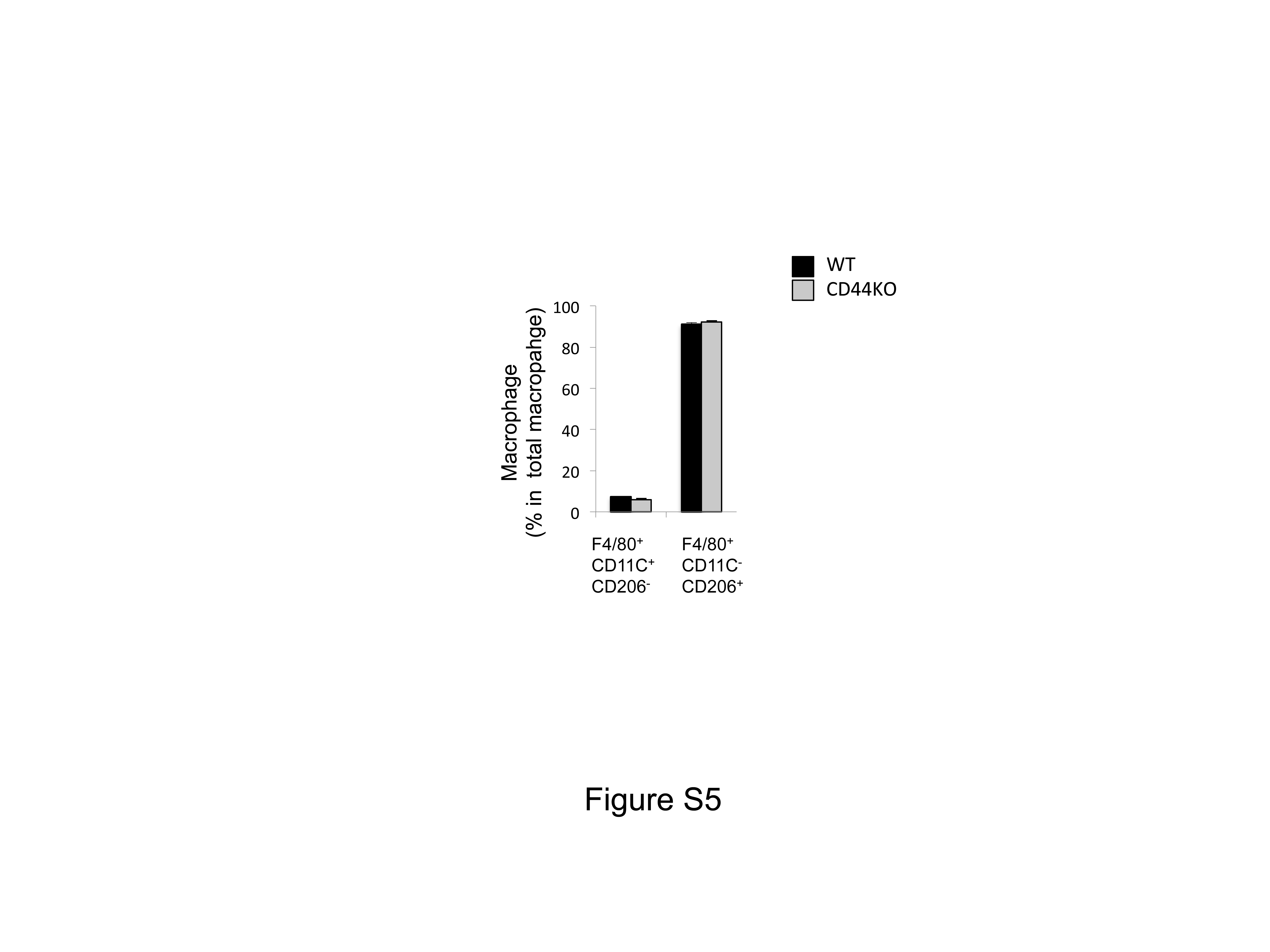

Supplement: Figure S5 — Macrophage populations of SVF cells from WAT of WT(ND) and CD44KO(ND) mice (n = 6 mice per each group) were examined by FACS. No difference was observed in the percentage of M1 and M2 macrophages between WT and CD44KO mice before HFD. (TIF) [file pone.0058417.s005.tif]

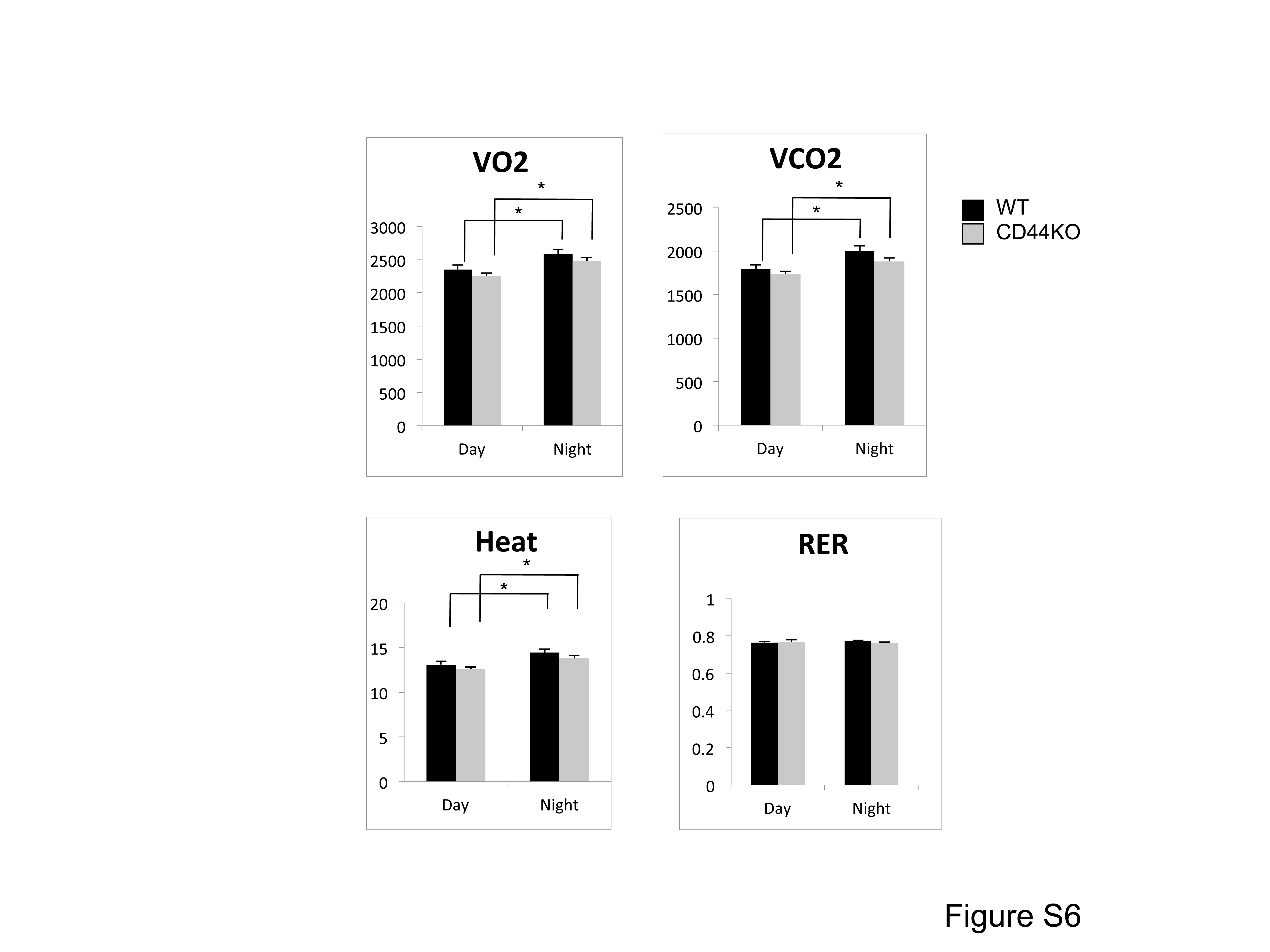

Supplement: Figure S6 — Oxygen consumption, CO2 production, Heat generation, and respiratory exchange ratio (RER) were analyzed in WT(HFD) and CD44KO(HFD) mice (n = 6 mice per each group) with a LabMaster system (TSE systems INC, Chesterfield, MO). (TIF) [file pone.0058417.s006.tif]
