# Supplementary material for: CD44 Plays a Critical Role in Regulating Diet-Induced Adipose Inflammation, Hepatic Steatosis, and Insulin Resistance
Source: PLoS One. 2013 Mar 7;8(3):e58417. doi: 10.1371/journal.pone.0058417 (PMC3591334; doi:10.1371/journal.pone.0058417)
Supplement: Table S1 — List of QRT-PCR primers. (DOCX) [file pone.0058417.s007.docx]

**Table S1.** List of QRT-PCR primers

| Gene | Forward primer | Reverse primer | Probe (5’-3-FAM 3’-TAMRA) |
| --- | --- | --- | --- |
| 18S | CGGCTACCACATCCAAGGAA | GCTGGAATTACCGCGGCT | TGCTGGCACCAGACTTGCCCTC |
| Atgl | ACCTGATGACCACCCTTTCCA | AACAAGCGGATGGTGAAGGAC |  |
| Ccl2 | TCTCTCTTCCTCCACCACCATG | GCGTTAACTGCATCTGGCTGA |  |
| Ccl7 | TGCTTTCAGCATCCAAGTGTG | TTGAAGATAACAGCTTCCCAGG |  |
| Ccr2 | CTCAAGCCACAGTTCCTTACATGAT | CCCTGGAAAGCAGAAGGAAAGATC |  |
| Ccr5 | CGAAAACACATGGTCAAACG | GTTCTCCTGTGGATCGGGTA |  |
| Cd11c | AAAATCTCCAACCCATGCTG | CACCACCAGGGTCTTCAAGT |  |
| Cd44 | GTCTGCATCGCGGTCAATAGTAG | TCCCATTGCCACCGTTGATC |  |
| Cd36 | TCATGCCAGTCGGAGACATG | TGTCTGTACACAGTGGTGCCTGT |  |
| Cidea | ATCACAACTGGCCTGGTTACG | TACTACCCGGTGTCCATTTCT |  |
| Cidec | CCTATGACCTGCACTGCTACAAG | CATGTAGCTGGAGGTGCCAAG |  |
| Cpt-1a | CCTGGGCATGATTGCAAAG | GGACGCCACTCACGATGTT |  |
| Dgat1 | GTGCACAAGTGGTGCATCAG | CAGTGGGATCTGAGCCATCA |  |
| Dgat2 | CAGCAAGAAGTTTCCTGGCAT | CCTCCCACCACGATGATGAT |  |
| F4/80 | ACCCTCCAGCACATCCAGCCA | TCACAGCCCGAGGGTGTCCA |  |
| Fasn | CCCTTGATGAAGAGGGATCA | CAAGGCGTTAGGGTTGACAT |  |
| FoxO1 | GCAGCCAGGCATCTCATAA | CCTACCATAGCCATTGCAGC |  |
| G6Pase | ATGAACATTCTCCATACTTTGGG | GACAGGGAACTGCTTTATTATAGG |  |
| Hsl | TTCGCCATAGACCCAGAGTTG | CGACAGCACCTCAATCTCAGTG |  |
| Il1rn | TGAGACGTTGGAAGGCAGTGGAAG | GCAGGCTGCCTCTGAAGCCATG |  |
| Il-6 | GAGAAAAGAGTTGTGCAATGGC | CCAGTTTGGTAGCATCCATCAT |  |
| Leptin | CCGTGGGTACACGCTTCGCT | GGATGCAGCCTTCACGGTGCT |  |
| Lipin1 | CCCTCGATTTCAACGTACCC | GCAGCCTGTGGCAATTCA |  |
| Mac-2 | ATGAAGAACCTCCGGGAAAT | TTAGATCATGGCGTGGTTAGC |  |
| Mmp12 | CCCACTTCGCCAAAAGGTTT | CATGAGCTCCTGCCTCACATC |  |
| Mogat1 | CCAGCGCAAAGGGTTTGTT | CACCAAAAGAAAATACTGGAACCA |  |
| My6d | TGTCACGTGTGCACCAACAGT | TTCCCATTCAGAGGCTCCACT |  |
| Opn | GAGTTTCCAGGTTTCTGATGAACA | TTAGACTCACCGCTCTTCATGTG |  |
| Pgc-1α | CCCTGCCATTGTTAAGACC | TGCTGCTGTTCCTGTTTTC |  |
| Srebp1-c | ACGGAGCCATGGATTGCAC | TGTCTCACCCCCAGCATAG |  |
| Tnfα | CAAAATTCGAGTGACAAGCCTG | CACTCCAGCTGCTCCTCCAC |  |
| Ucp-3 | GACTATGGATGCCTACAGAACC | ACTCCAGCAACTTCTCCTTG |  |
| Tlr8 | GGCACAACTCCCTTGTGATT | TGTTGTTTGGCATTGTGGTT |  |
