# Supplementary material for: CD44 Plays a Critical Role in Regulating Diet-Induced Adipose Inflammation, Hepatic Steatosis, and Insulin Resistance
Source: PLoS One. 2013 Mar 7;8(3):e58417. doi: 10.1371/journal.pone.0058417 (PMC3591334; doi:10.1371/journal.pone.0058417)
Supplement: Table S2 — Partial list of genes reduced or increased in WAT of CD44KO(HFD) mice compared to WT(HFD) mice. (DOCX) [file pone.0058417.s008.docx]

**Table S2.** Partial list of genes reduced or increased in WAT of CD44KO(HFD) mice compared to WT(HFD) mice.

| Functional Category | GeneBank Accession # | Gene Symbol | Gene description | Fold Change (KO/WT) |
| --- | --- | --- | --- | --- |
| Inflammation associated genes |  |  |  |  |
| *Chemokine* |  |  |  |  |
|  | NM_145700 | Ccrl1 | chemokine (C-C motif) receptor-like 1 | -4.57 |
|  | NM_019568 | Cxcl14 | chemokine (C-X-C motif) ligand 14 | -2.49 |
|  | NM_009912 | Ccr1 | chemokine (C-C motif) receptor 1 | -2.38 |
|  | NM_009139 | Ccl6 | chemokine (C-C motif) ligand 6 | -1.75 |
|  | NM_009916 | Ccr5 | chemokine (C-C motif) receptor 5 | -1.50 |
|  | NM_013655 | Cxcl12 | chemokine (C-X-C motif) ligand 12 | 1.69 |
| *Interleukin & Receptor* | |  |  |  |
|  | NM_010559 | Il6ra | interleukin 6 receptor, alpha | -2.34 |
|  | AK018347 | Il10rb | interleukin 10 receptor, beta | -2.32 |
|  | NM_010552 | Il17a | interleukin 17a | -2.30 |
|  | NM_133775 | Il33 | interleukin 33 | -2.24 |
|  | NM_001039701 | Il1rn | interleukin receptor 1 antagonist | -2.06 |
|  | NM_008366 | Il2 | interleukin 2 | -2.02 |
|  | D13695 | Il1rl1 | interleukin 1 receptor-like 1 | -1.94 |
| *Immune response* |  |  |  |  |
|  | AK045226 | Cd44 | CD44 antigen | -26.05 |
|  | NM_010781 | Tpsb2 | tryptase beta 2 | -14.28 |
|  | NM_031187 | Tpsab1 | tryptase alpha/beta 1 | -12.06 |
|  | AY151141 | Igl-V1 | immunoglobulin lambda chain, variable 1 | -9.26 |
|  | NM_178372 | Prss34 | protease, serine, 34 | -8.05 |
|  | NM_012034 | Tpsg1 | tryptase gamma 1 | -6.50 |
|  | NM_009857 | Cd8a | Cd8 antigen, alpha chain | -4.37 |
|  | M17534 | Cd8b | Cd8 antigen, beta chain | -3.51 |
|  | NM_011414 | Slpi | secretory leukocyte peptidase inhibitor | -3.76 |
|  | NM_019563 | Cited4 | Cbp/p300-interacting transactivator 4 | -3.39 |
|  | NM_013487 | Cd3 | Cd3 antigen | -2.99 |
|  | NM_016711 | Tmod2 | tropomodulin 2 | -2.26 |
|  | NM_013529 | Gfpt2 | glutamine fructose-6-phosphate transaminase 2 | -2.17 |
|  | AK040902 | Rnf19b | Ring finger protein 19B | -2.16 |
|  | NM_009099 | Trim30 | tripartite motif-containing 30 | -2.08 |
|  | NM_031195 | Msr1 | macrophage scavenger receptor | -2.01 |
|  | AK048506 | Nfkb1 | NF-k light polypeptide gene enhancer in B-cells 1 | -1.96 |
|  | BY667523 | Hif3a | Hypoxia inducible factor 3, alpha subunit | -1.90 |
|  | NM_172659 | Slc2a6 | solute carrier family 2, member 6 | -1.90 |
|  | NM_145634 | Cd300lf | CD300 antigen like family member F | -1.87 |
|  | XM_135029 | Fam46a | family with sequence similarity 46, member A | -1.73 |
|  | NM_133212 | Tlr8 | toll-like receptor 8 | -1.64 |
|  | NM_028270 | Aldh1b1 | aldehyde dehydrogenase 1 family, member B1 | -1.64 |

| Functional Category | GeneBank Accession # | Gene Symbol | Gene description | Fold Change (KO/WT) |
| --- | --- | --- | --- | --- |
|  | NM_176860 | Ubash3b | ubiquitin associated and SH3 domain containing, B | -1.61 |
|  | NM_008987 | Ptx3 | pentraxin related gene | -1.54 |
|  | NM_181397 | Rftn1 | raftlin lipid raft linker 1 | -1.40 |
|  | NM_025729 | Tab3 | TGF-beta activated kinase 1 binding protein 3 | 1.43 |
|  | NM_028980 | Ppp4r4 | protein phosphatase 4, regulatory subunit 4 | 1.60 |
|  | NM_178934 | Slc2a12 | solute carrier family 2, member 12 | 2.02 |
| *Cell matrix and surface* | |  |  |  |
|  | NM_025622 | Lgals2 | lectin, galactose binding, soluble 2 | -3.59 |
|  | NM_010689 | Lat | linker for activation of T cells | -3.27 |
|  | NM_008605 | Mmp12 | matrix metalloproteinase | -2.40 |
|  | AK041322 | Cd28 | CD28 antigen | -2.35 |
|  | AK037794 | Itga4 | integrin alpha 4 | -2.34 |
|  | NM_008401 | Itgam | integrin alpha M (Mac-1, Cd11b) | -1.99 |
|  | NM_008401 | Itgax | integrin alpha X (Cd11c) | -1.99 |
|  | NM_010130 | Emr1 | EGF-like module receptor (F4/80) | -1.94 |
|  | NM_010493 | Icam1 | intercellular adhesion molecule 1 | -1.80 |
|  | AK053377 | Itga1 | integrin alpha 1 | 1.69 |
|  | XM_354704 | Igh-1a | Similar to Ig gamma-2A chain C secreted form | -16.62 |
| Lipid accumulation associated genes |  |  |  |  |
|  | NM_017399 | Fabp1 | fatty acid binding protein 1 | 2.88 |
|  | NM_007703 | Elovl3 | elongation of long chain fatty acids 3 | 2.32 |
|  | AK080374 | Fasn | fatty acid synthase | 2.27 |
|  | NM_130450 | Elovl6 | elongation of long chain fatty acids 6 | 2.26 |
|  | NM_177448 | Mogat2 | monoacylglycerol O-acyltransferase 2 | 2.00 |
|  | NM_013459 | Cfd | complement factor D (adipsin) | 1.77 |
|  | AK036010 | Vldlr | very low density lipoprotein receptor | 1.71 |
|  | AK052945 | Fitm2 | fat storage-inducing transmembrane protein 2 | 1.71 |
|  | NM_029001 | Elovl7 | ELOVL family member 7 | -2.72 |
